# Induction of Cell-Cell Fusion by Ebola Virus Glycoprotein: Low pH Is Not a Trigger

**DOI:** 10.1371/journal.ppat.1005373

**Published:** 2016-01-05

**Authors:** Ruben M. Markosyan, Chunhui Miao, Yi-Min Zheng, Gregory B. Melikyan, Shan-Lu Liu, Fredric S. Cohen

**Affiliations:** 1 Rush University Medical Center, Department of Molecular Biophysics and Physiology, Chicago, Illinois, United States of America; 2 University of Missouri School of Medicine, Bond Life Sciences Center, Department of Molecular Microbiology and Immunology, Columbia, Missouri, United States of America; 3 Emory University Medical School, Department of Pediatrics, Infectious Diseases, Atlanta, Georgia, United States of America; Mount Sinai School of Medicine, UNITED STATES

## Abstract

Ebola virus (EBOV) is a highly pathogenic filovirus that causes hemorrhagic fever in humans and animals. Currently, how EBOV fuses its envelope membrane within an endosomal membrane to cause infection is poorly understood. We successfully measure cell-cell fusion mediated by the EBOV fusion protein, GP, assayed by the transfer of both cytoplasmic and membrane dyes. A small molecule fusion inhibitor, a neutralizing antibody, as well as mutations in EBOV GP known to reduce viral infection, all greatly reduce fusion. By monitoring redistribution of small aqueous dyes between cells and by electrical capacitance measurements, we discovered that EBOV GP-mediated fusion pores do not readily enlarge—a marked difference from the behavior of other viral fusion proteins. EBOV GP must be cleaved by late endosome-resident cathepsins B or L in order to become fusion-competent. Cleavage of cell surface-expressed GP appears to occur in endosomes, as evidenced by the fusion block imposed by cathepsin inhibitors, agents that raise endosomal pH, or an inhibitor of anterograde trafficking. Treating effector cells with a recombinant soluble cathepsin B or thermolysin, which cleaves GP into an active form, increases the extent of fusion, suggesting that a fraction of surface-expressed GP is not cleaved. Whereas the rate of fusion is increased by a brief exposure to acidic pH, fusion does occur at neutral pH. Importantly, the extent of fusion is independent of external pH in experiments in which cathepsin activity is blocked and EBOV GP is cleaved by thermolysin. These results imply that low pH promotes fusion through the well-known pH-dependent activity of cathepsins; fusion induced by cleaved EBOV GP is a process that is fundamentally independent of pH. The cell-cell fusion system has revealed some previously unappreciated features of EBOV entry, which could not be readily elucidated in the context of endosomal entry.

## Author Summary

The devastation and transmissibility of Ebola virus (EBOV) are well known. However, the manner in which EBOV enters host cells through endosomal membrane remains elusive. Here, we have developed a convenient experimental system to mimic EBOV fusion in endosomes: cells expressing the fusion protein of EBOV, GP, on their surface are fused to target cells. This system exhibits the known key properties of EBOV fusion. We show that the pH-dependence of EBOV fusion is caused by the pH-dependence of cathepsins, proteases known to cleave EBOV GP into a fusion-competent form. We demonstrate that the fusion activity of this cleaved form is independent of pH. We further show that the enlargement of the fusion pore created by EBOV GP is unusually slow in reaching sizes necessary to pass EBOV’s genome—this is atypical of virally created fusion pores. This cell-cell fusion system should provide a useful platform for developing drugs against EBOV infection.

## Introduction

Ebola virus (EBOV) outbreaks continually occur and up to 90% of those infected die; currently there are no approved vaccines or antiviral therapeutics against the virus [1,2]. EBOV initiates infection by fusion from within endosomes. Experimentally, endosomal interiors are difficult to control, but systems that track the entry of several other viruses into cells have been developed and employed [3,4,5,6]. Historically, these methods have relied on fusion of infectious virus or pseudovirus within cells; cell-cell fusion has not been among the systems in use for EBOV. It is surprising that a cell-cell fusion system has not been developed, as the processing of the Ebola fusion protein, GP, and other conditions necessary for fusion have been elaborated [7]. (Some years ago there was an isolated report of EBOV GP-mediated cell-cell fusion, but this study has not been followed up by any other laboratory, including the original [8]). Cell-cell fusion has several important advantages over intracellular fusion assays, including complete control of the aqueous solution bathing the ectodomain of the fusion protein. In the present study we describe a direct and sensitive system to measure EBOV GP-mediated cell-cell fusion with high time resolution, thereby providing fusion kinetics. The system exhibits the well-known central properties of EBOV entry, providing strong support for the utility of the cell-cell fusion system to explore mechanisms of EBOV entry that are not possible or practical with whole infectious virus.

EBOV GP is a prototypic class I viral fusion protein [9]. It is synthesized as a homotrimer; each monomer is cleaved into GP1-GP2 subunits by proteases within the Golgi apparatus [10,11]. The GP1 subunit is responsible for binding to the intracellular receptor Niemann Pick type C1, (NPC1) and possibly to other molecules [12], and the GP2 subunit is responsible for membrane fusion [13,14,15,16,17,18,19]. The two subunits of each monomer remain linked through a disulfide bond and a multitude of weak interactions [9,20,21,22]. After endocytosis of the virus, the GP1 subunit is cleaved by the endosomal proteases cathepsin B and/or L [7,23,24,25,26], while remaining attached to GP2 [9], and then binds to NPC1 [14,15]. The low pH within endosomes is necessary for viral fusion. But it has not been known whether low pH directly triggers fusion by causing conformational changes in GP or whether it augments fusion by increasing the activities of the cathepsins [7,25].

After developing our system, we discovered that an EBOV GP-induced fusion pore that connects two plasma membranes does not readily enlarge over time, in contrast to the pores of other viral fusion proteins. This anomalous lack of growth may be the reason cell-cell fusion has not been successfully observed in many prior attempts that used less sensitive assays to detect fusion.

On the question of low pH, we found that activation of cathepsins by acidity is the sole cause for augmentation of fusion: if EBOV GP on the cell surface is artificially cleaved by thermolysin in the presence of cathepsin inhibitors, the extent of fusion is independent of pH.

## Results

### EBOV GP mediates cell-cell fusion

**Fig 1:**
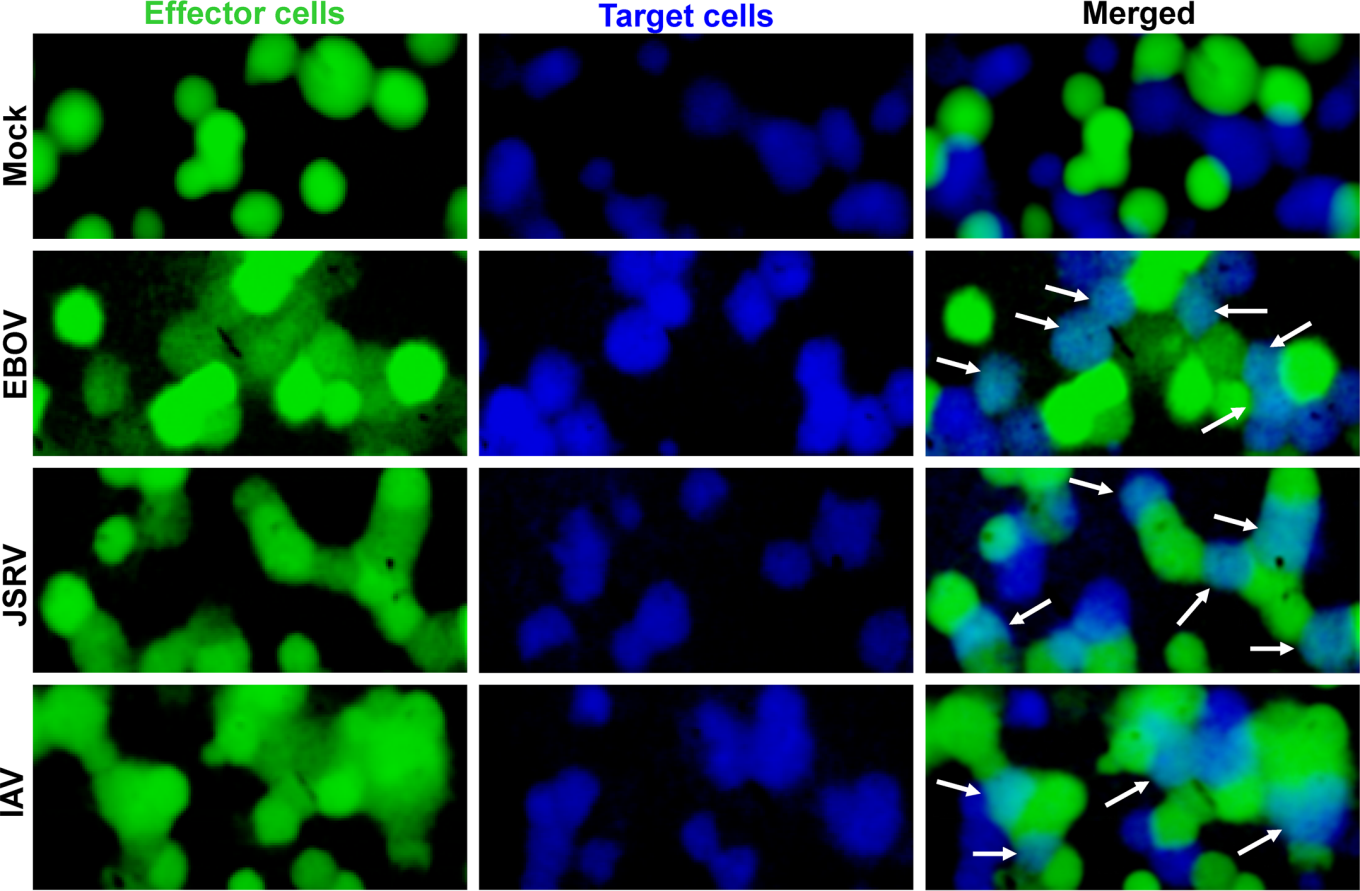
Images of fused effector and target cells. Effector (COS7) cells were loaded with calcein-AM (column 1, green), target cells were loaded with CMAC (column 2, blue) and both dyes are shown in column 3 (merged). The viral proteins expressed by transfecting effector cells are shown to the right of the images. Cells expressing EBOV GP were treated with 200 μg/ml thermolysin for 20 min; fusion was augmented with a 10-min pH 5.7 pulse. For cells expressing JSRV Env, a 10-min pH 5.0 pulse was used to trigger fusion. Effector cells expressing influenza virus (IAV) HA were treated with trypsin and neuraminidase as described [28], bound to HEK 293T cells, and fusion was triggered with a 10-min pH 4.8 pulse. For mock-transfected effector cells, a 10-min pH 5.7 pulse was employed. Fused cells are marked by arrowheads. For this set of experiments, the extent of fusion 1 hr after reneutralization was about 80% for EBOV GP, 50% for JSRV Env, and 70% for IAV HA.

We utilize fluorescent dye spread assays to monitor cell-cell fusion. Effector COS7 cells transfected to express EBOV GP were loaded with calcein-AM (CaAM, green) and pretreated with thermolysin (Th) and. It has been shown that thermolysin treatment cleaves GP1 on the viral membrane into a fusion-competent 18–19 kDa subunit [9,23,24]. Within the laboratory, thermolysin is therefore often used in lieu of membrane-bound cathepsins to cleave the GP1 subunit into a fusion-competent form. The COS7 cells were bound to 293T target cells that were either unlabeled or, for purposes of microscopic identification, loaded with the aqueous dye CMAC (blue). We lowered the external pH for 10 min at room temperature, reneutralized, raised temperature to 37°C, and monitored dye spread at various times. We tracked the transfer of calcein between cells to quantify the extents of fusion; CMAC was used solely to identify the target cells. The fraction of cells that were stained by both calcein and CMAC, 2 hr after a 10-min low pH pulse, was comparable for cell-cell fusion mediated by EBOV GP, by Jaagsiekte sheep retrovirus (JSRV) Env, and influenza A virus (IAV) hemagglutinin (HA)—all requiring low pH for fusion to proceed (Fig 1). Fusion did not occur for effector cells that were mock transfected, establishing that CaAM transferred only due to fusion (top row).

**Fig 2:**
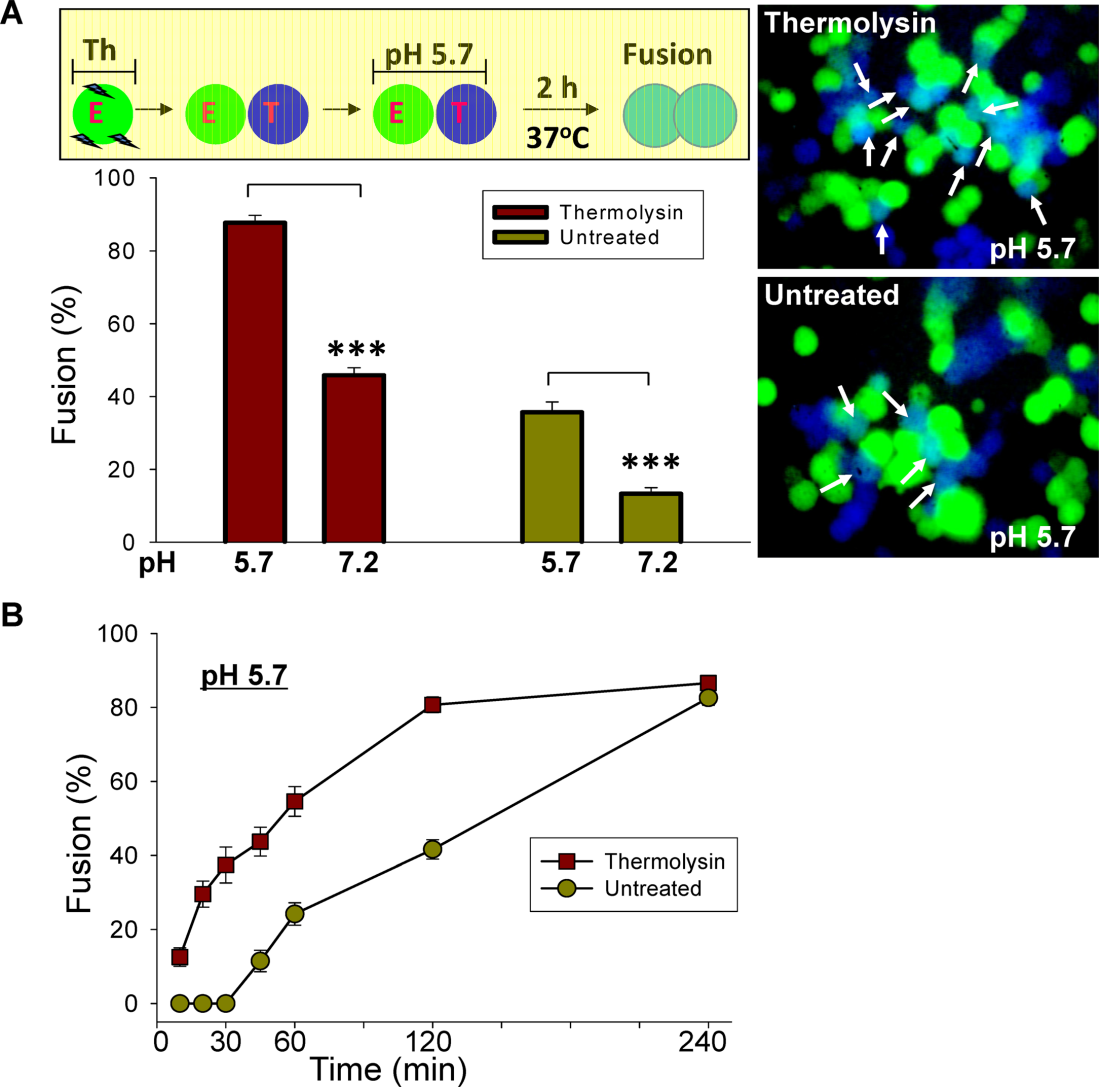
Thermolysin treatment results in greater extents of fusion between cells. **(A)** schematic of the experimental protocol is shown above the bar graph. E, effector cells; T, target cells, Th, thermolysin. Bar graph: Fusion of thermolysin-treated effector cells expressing EBOV GP (columns 1 and 2, dark red) was greater than for untreated cells (columns 3 and 4, dark yellow). For both thermolysin-treated and non-treated cells, a 10-min pH 5.7 pulse applied at room temperature augmented fusion, measured after an additional 2 h incubation at neutral pH. For each condition, at least 7 experiments were performed. Typical images used to obtain the data of the bar graph are shown on the right: in top images, cells were treated with 200 μg/ml thermolysin; in bottom images, cells were not treated. Cells that have fused are marked by arrows. **(B)** The kinetics of fusion for thermolysin-treated (dark red squares) and untreated (dark yellow circles) effector cells. Cleaving EBOV GP by thermolysin speeds fusion kinetics, but extents of fusion are the same for treated and untreated cells after a pH 5.7 pulse at 10°C is followed by a 4 h reneutralization. * p <0.05; *** p < 0.001.

It is often thought that EBOV fusion requires acidic pH [7,25,27]. But we found that thermolysin-treated effector cells expressing EBOV GP also fused to target cells at neutral pH (7.2) (Fig 2A, bar 2), albeit to a smaller extent than occurred 2 hrs after a 10 min exposure to an acidic pH of 5.7 at room temperature (bar 1). Representative images for dye transfer are shown to the right of the bar graphs (Fig 2A). In its natural cellular setting, EBOV GP is cleaved not by thermolysin but by endosomal cathepsins B and L. In measuring fusion without prior thermolysin treatment of effector cells, we found that fusion still occurred, albeit to smaller extents (Fig 2A, bars 3 and 4). Again, a 10-min acidification (Fig 2A, bar 3) led to greater amounts of fusion than occurred at neutral pH when measured after a 2-h reneutralization (Fig 2A, bar 4). Mock transfected effector cells, with or without thermolysin treatment, did not support any dye transfer at neutral or low pH, verifying that fusion required EBOV GP (e.g., see Fig 1).

The observed differences in extents of fusion between cells that were treated with thermolysin and those not were eliminated by long times of incubation after reneutralization (Fig 2B). When EBOV GP was not cleaved by thermolysin, there was a 30 min latency between the fusion trigger (acidification and raising temperature from 10°C to 37°C, Fig 2B, dark yellow circles) and the occurrence of fusion. There was no latency when thermolysin cleaved the protein (dark red squares, same fusion trigger as for dark yellow circles), suggesting that the 30 min latency when thermolysin was not used was due to the time it takes for a sufficient number of copies of cleaved GP to accumulate at a potential fusion site. The extent of fusion for non-treated effector cells (dark yellow circles) 2 hrs after reneutralization was almost equal to that observed after a 1 hr reneutralization for thermolysin-treated cells. But 4 hrs after a pH 5.7 pulse, the extent of fusion was independent of whether EBOV GP was cleaved by thermolysin. The kinetic difference is, to a large extent, likely due to the ~30 min latency until fusion occurs. The slopes of the linear portion for rates of fusion are comparable, suggesting that, after the latency, the kinetics of fusion are the same at low and neutral pH. The latency for EBOV GP-mediated fusion is much longer than for some viral fusion proteins, such as IAV HA [28], but comparable to others, such as HIV Env in some studies [29]. We thus tested, at various times, whether some of the cell pairs that had not yet fully fused had hemifused: the addition of 0.5 mM CPZ to cell pairs ruptures hemifusion diaphragms that have formed between cell pairs, and this is a standard means to test for hemifusion [30,31,32]. We used thermolysin-treated effector cells to maximize cleavage of EBOV GP and found that adding CPZ either 30, 45, or 60 min after reneutralization did not induce any dye spread above that already observed, strongly indicating that a negligible percentage of cells were hemifused, but not fused, at any given time.

**Fig 3:**
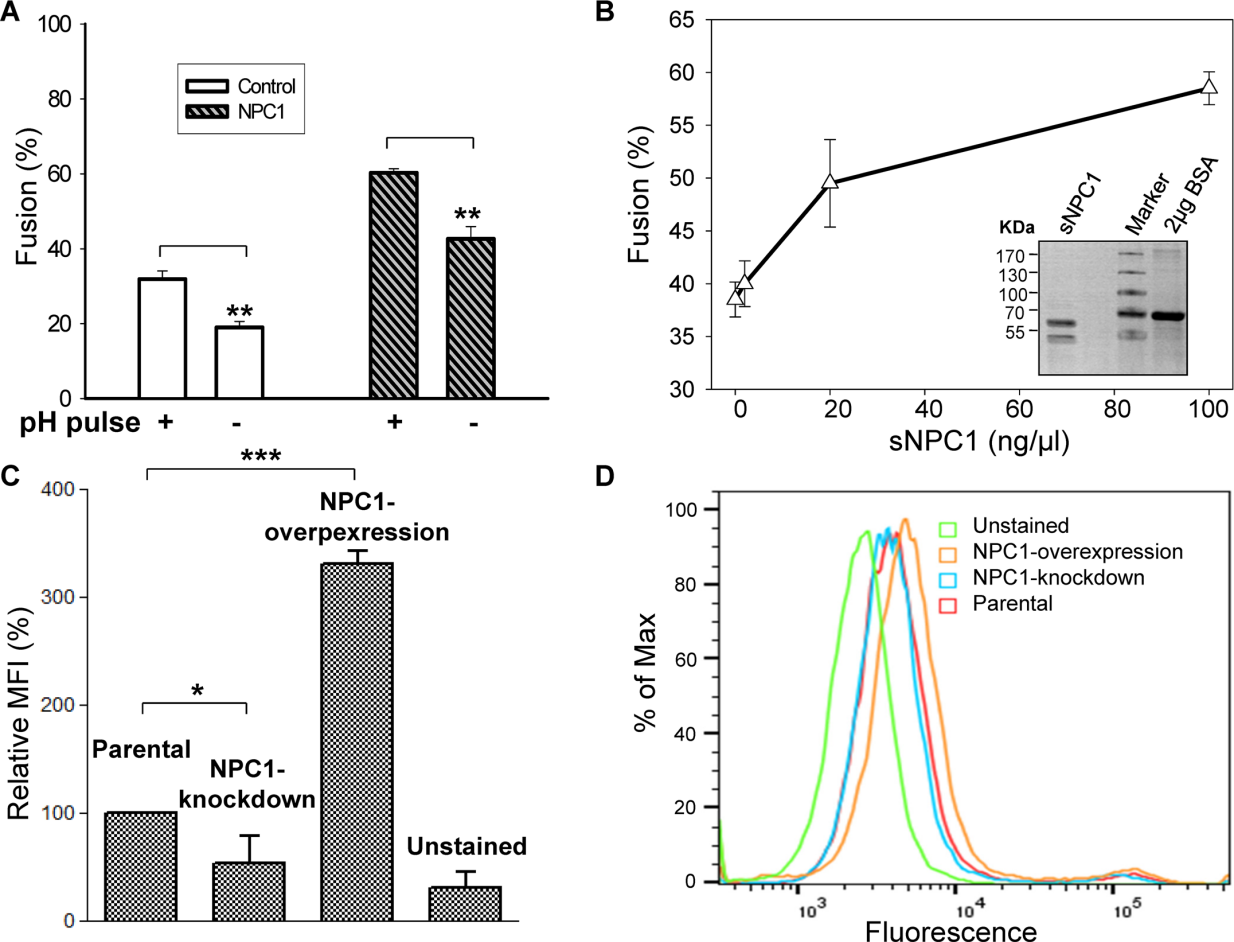
Extents of fusion increased by overexpressing the receptor for EBOV GP. **(A)** Overexpression of NPC1 (second set of two bars) led to greater fusion with effector cells than did mock-transfected target cells (first set of bars). For these experiments, the effector cells were not thermolysin-treated (i.e., these experiments relied on endogenous levels of GP cleavage). For each set of experiments, a 10-min pH 5.7 pulse (labeled “pulse pH +”) led to more fusion than when pH was never lowered (-). For each condition, n = 4. **(B)** The addition of sNPC1 to the external solution leads to a greater extent of fusion. *Inset*: Coomassie staining verification of sNPC1. BSA serves as a loading control. **(C)** Reducing and increasing the expression levels of NPC1 results in changes in the amount of NPC1 on the plasma membrane. **(D)** Fluorescence profiles of NPC1 from flow cytometry.

NPC1 is an intracellular receptor for EBOV GP [14,33]. We compared extents of fusion for target parental HEK 293T cells versus target HEK 293T cells that stably overexpressed NPC1. Effector cells that were not treated with thermolysin yielded fusion at pH 7.2 (Fig 3A, bar 2), and a greater extent of fusion after a 10-min acidic pH 5.7 pulse (Fig 3A, bar 1). The extent of calcein spread was greater for target cells overexpressing NPC1 (Fig 3A, bars 3 and 4) than for parental 293T cells (Fig 3A, bars 1 and 2) for matching conditions. Fusion was still pH-dependent for target cells overexpressing NPC1: calcein spread was greater 2 hr after a 10-min pH 5.7 pulse (Fig 3A, bar 3) than in the absence of the pulse (Fig 3A, bar 4). We confirmed that fusion was dependent on the presence of NPC1 by generating and purifying a recombinant soluble protein consisting of domain C of NPC1 fused to GST (denoted sNPC1). The purity and size of sNPC1 was confirmed (Fig 3B, inset). We added sNPC1 to the external solution and found that the extent of fusion increased monotonically with the amount of sNPC1 added (Fig 3B), in accord with the prior demonstration that by binding NPC1, EBOV GP undergoes conformational changes favorable for fusion [18]. The augmentation of fusion by sNPC1 indicated that, although there was a sufficient amount of NPC1 on cell surfaces to stimulate fusion, this amount was relatively small and fusion was consequently limited.

NPC1 is an endosomal protein [34], but a small fraction of NPC1 may be present on the plasma membrane of a cell. We assessed this possibility by using flow cytometry to measure binding with an antibody against NPC1 (from LifeSpan Biosciences) on parental 293 cells; shRNA that targeted NPC1 was stably expressed in one set of these 293 cells, and NPC1 was overexpressed in another set (Fig 3C and 3D). The level of binding of the secondary FITC-labeled antibody against endogenous NPC1 (as measured by mean fluorescence intensity, MFI) was 3-fold greater than in the absence of the primary Ab (Fig 3C, bar 1 vs. bar 4, and Fig 3D). Expression was reduced for cells in which NPC1 was knocked down by shRNA (bar 2), and was greater for cells in which NPC1 was overexpressed (bar 3). These results demonstrate that copies of NPC1 reside in the plasma membrane of the 293 cells we used as targets in cell-cell fusion experiments.

EBOV GP is certainly cleaved within endosomes as part of viral infection [26]. Because we observed cell-cell fusion at acidic pH without adding thermolysin, it is extremely likely that a fraction of GP on the cell surface was cleaved into a fusion-competent form. An antibody that only recognizes cleaved GP has not been reported, so we had to devise an alternate means to quantitatively measure the extent of cleavage. We were able to distinguish between the two forms of GP by using the property that NPC1 binds to cleaved, but not uncleaved, EBOV GP. We used a sNPC1 to examine cleaved GP by flow cytometry; in parallel, we measured the total amount of GP on cell surfaces by using an anti-FLAG antibody that bound to the FLAG tag on our GP construct. We also created a GP construct that was intrinsically more likely to be cleaved on the cell surface: we inserted the furin recognition site RRKR at amino acids 203–206 of GP1 (referred to as GP^furin^), the putative cleavage site for CatL in GP1 [16,35]. We reasoned that because exogenous expression of furin facilitates cleavage at this inserted site, generating the fusion-active 18–19 kDa subunit, the extent of cleavage of GP on the plasma membrane as well as the extent of cell-cell fusion would be greater for this construct than for WT.

**Fig 4:**
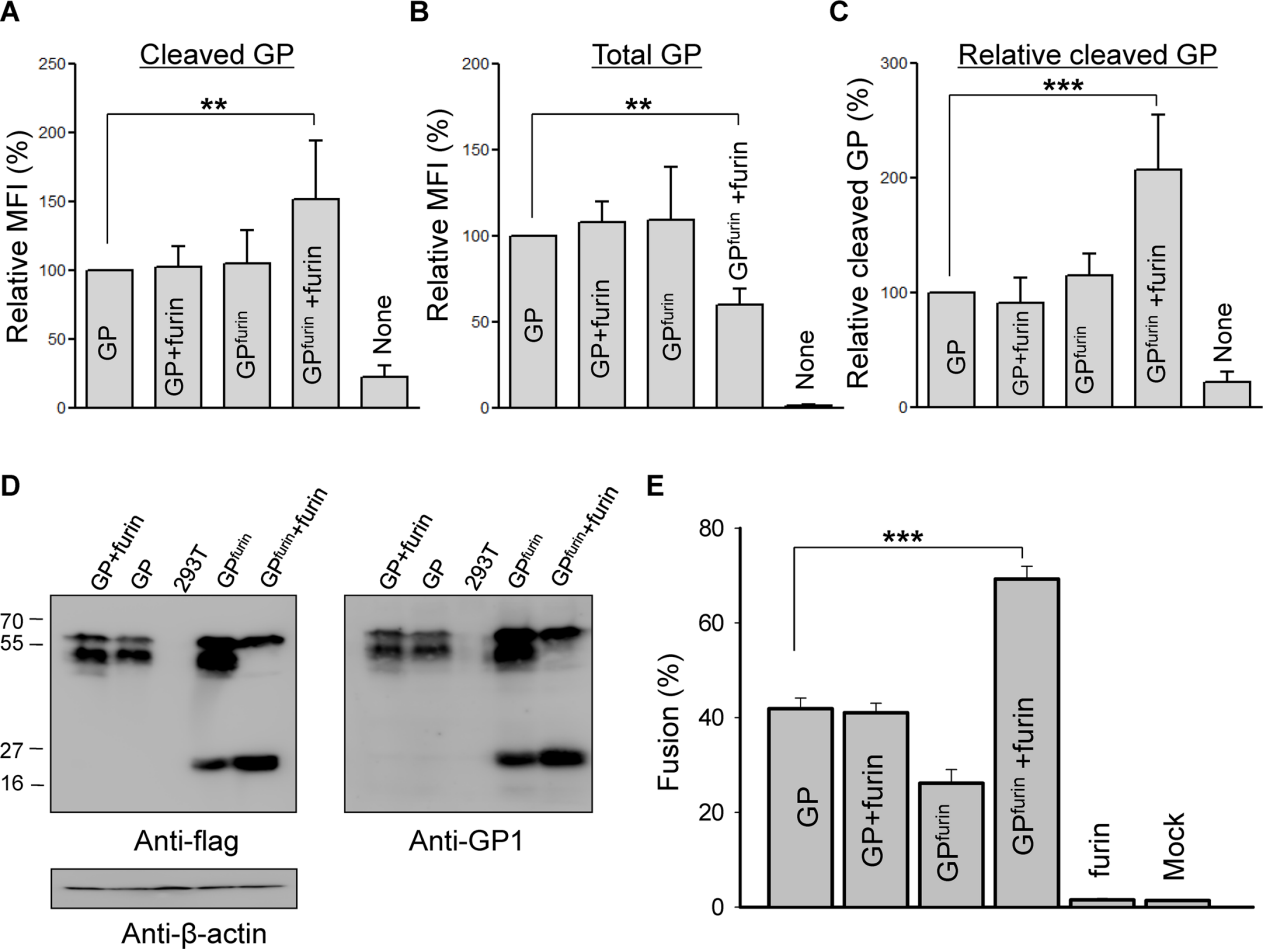
Detection of cleaved EBOV GP on the cell surface. **(A)** The amount of cleaved GP or GP^furin^ on the cell surface with and without cotransfection of furin was determined by using sNPC1. Mean fluorescence intensity (MFI) was acquired by flow cytometry. Relative MFI was calculated by setting the WT GP without furin to 100. “None”: 293T cells that were not transfected with GP. Averages with standard deviations of at least 3 independent experiments are shown in each bar. **(B)** The expression of total GP, cleaved and uncleaved, on the cell surface was determined using an anti-FLAG antibody; in parallel, the same number of transfected cells were employed to measure cleaved GP using sNPC1. Relative MFI values are shown by setting the WT GP without furin to 100. “None”: 293T cells not expressing GP. **(C)** Relative cleaved GP on the plasma membrane measured in (A) was normalized by total GP measured in (B). **(D)** Western blots demonstrating cleavage of GP in the cell lysate of transfected cells used in panels **(A)**, **(B)**, and **(C). (E)** Extents of cell-cell fusion using the transfection protocols of (A), (B), and (C).

We experimentally confirmed our expectations: We determined the amount of cleaved GP on the cell surface by adding sNPC1 (fused with GST) to cells expressing either EBOV GP or GP^furin^, and measuring their binding to an anti-GST antibody. This antibody was detected by a FITC-conjugated secondary antibody (Fig 4). The fraction of WT GP cleaved on parental cells (Fig 4A, bar 1) was the same for cells that were transfected with both GP and furin (bar 2). The specificities of sNPC1 and antibody binding were confirmed by the 4–5 fold higher fluorescence than was seen for cells that did not express GP (bar 5). It is notable that cotransfection of cells by GP^furin^ and furin resulted in greater cleavage (bar 4 vs bar 3). We found that the expression of total WT GP as measured by the anti-FLAG antibody was not significantly altered by coexpression of furin (Fig 4B, columns 1 and 2), but cells that coexpressed GP^furin^ and furin consistently showed a decreased total GP (compare bar 3 and 4), possibly due to non-specific degradation of GP^furin^. To determine the relative percentage of cleaved GP, we normalized cleaved GP by total GP. (These are relative and not absolute percentages because different antibodies were used to detect cleaved vs. total GP.) We found that a higher percentage of GP on the plasma membrane was cleaved for cells coexpressing GP^furin^ and furin than for cells expressing WT GP or GP^furin^ alone (Fig 4C). Western blot analyses, using an anti-FLAG or an anti-GP1 antibody (kind gift of James Cunningham), showed that the addition of furin increased the amount of cleaved GP^furin^ construct as compared to GP^furin^ alone (Fig 4D, lanes 4 and 5 in left and right panels). Furin did not cleave any WT GP (lanes 1). We used these constructs to verify that an increased cleavage of EBOV GP led to a greater extent of fusion (Fig 4E). Cotransfecting cells with GP and furin (bar 2) led to the same extent of fusion as did transfection of GP alone (bar 1). In contrast, cotransfecting with GP^furin^ and furin led to more fusion (bar 4) than transfecting only GP^furin^ (bar 3). Control experiments of transfecting only furin showed that furin, per se, did not promote fusion (bar 5). These experiments, taken together, establish that EBOV GP does appear on the cell surface, that some of it is cleaved, and that for the GP^furin^ construct, cleavage is augmented by coexpression of furin.

**Fig 5:**
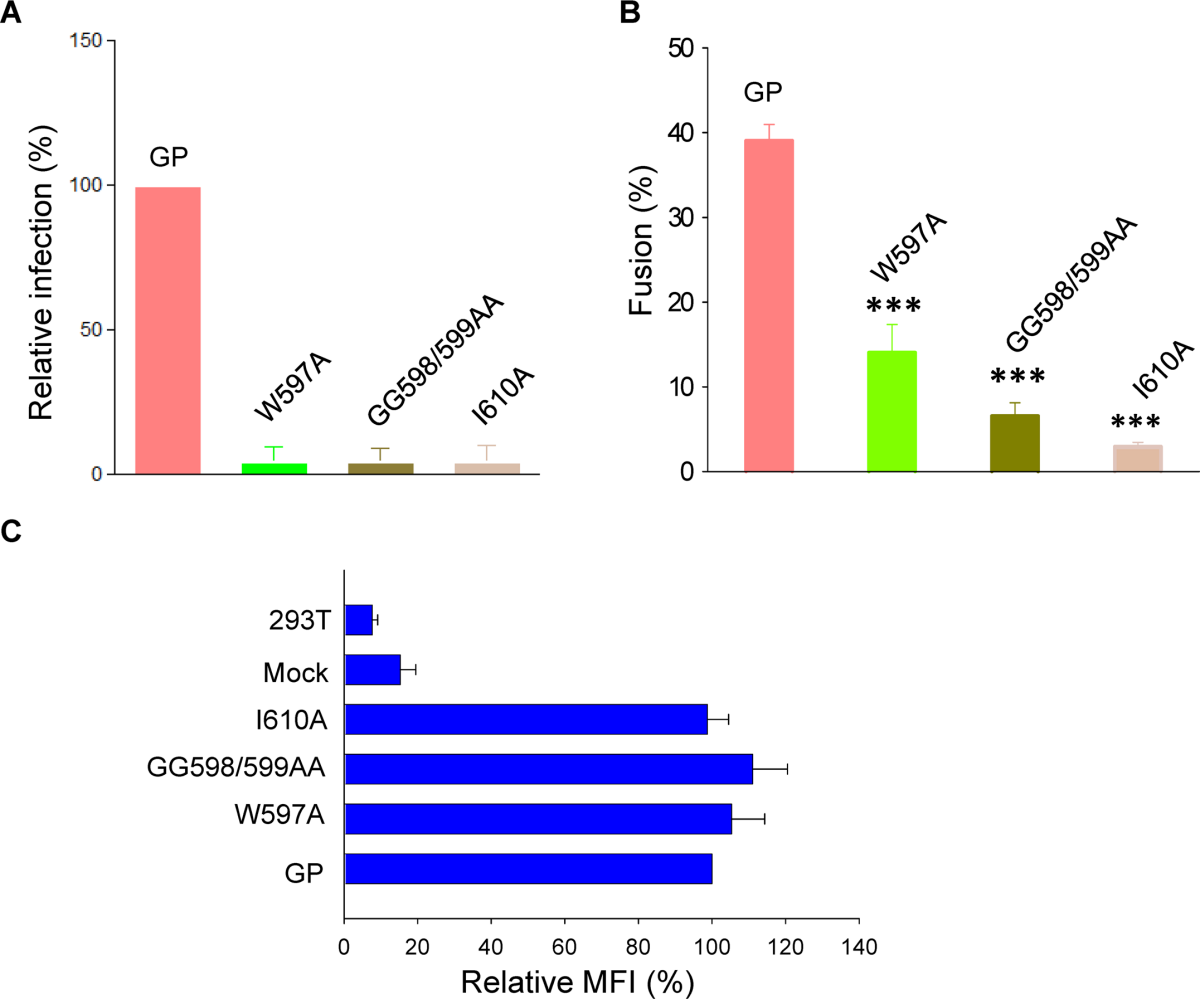
Reduced infection caused by mutations within EBOV GP correlates with reduced fusion. **(A)** The reduction in retroviral MLV pseudotyped infection is shown for a series of EBOV GP mutants. **(B)** The mutations that resulted in reduced infection also led to reduced cell-cell fusion. **(C)** Each of the mutants was expressed well on the cell surface as determined by flow cytometry using an anti-FLAG antibody.

To further confirm that the observed fusion was indeed mediated by EBOV GP, we utilized mutations that had previously been shown to greatly reduce viral infection [36]. We used MLV pseudovirus expressing GP, and observed that, indeed, the level of infection caused by the point mutant W597A (Fig 5, bar 2), the double mutant G598A/G599A (bar3), and the point mutant I610A (bar 4) were all substantially less than for WT GP (Bar 1). We then measured the extents of cell-cell fusion mediated by each of the mutant proteins. The extent of fusion in absence of thermolysin treatment supported by all three of the mutants (Fig 5B, bars 2, 3, and 4) was much less than for WT GP (bar 1). Flow cytometry measurements, using the same cells as for fusion experiments, showed that each of the mutant GPs was well expressed on the cell surface (Fig 5C). These experiments provide support that reduced infectivity by EBOV correlates with reduced GP-mediated fusion.

**Fig 6:**
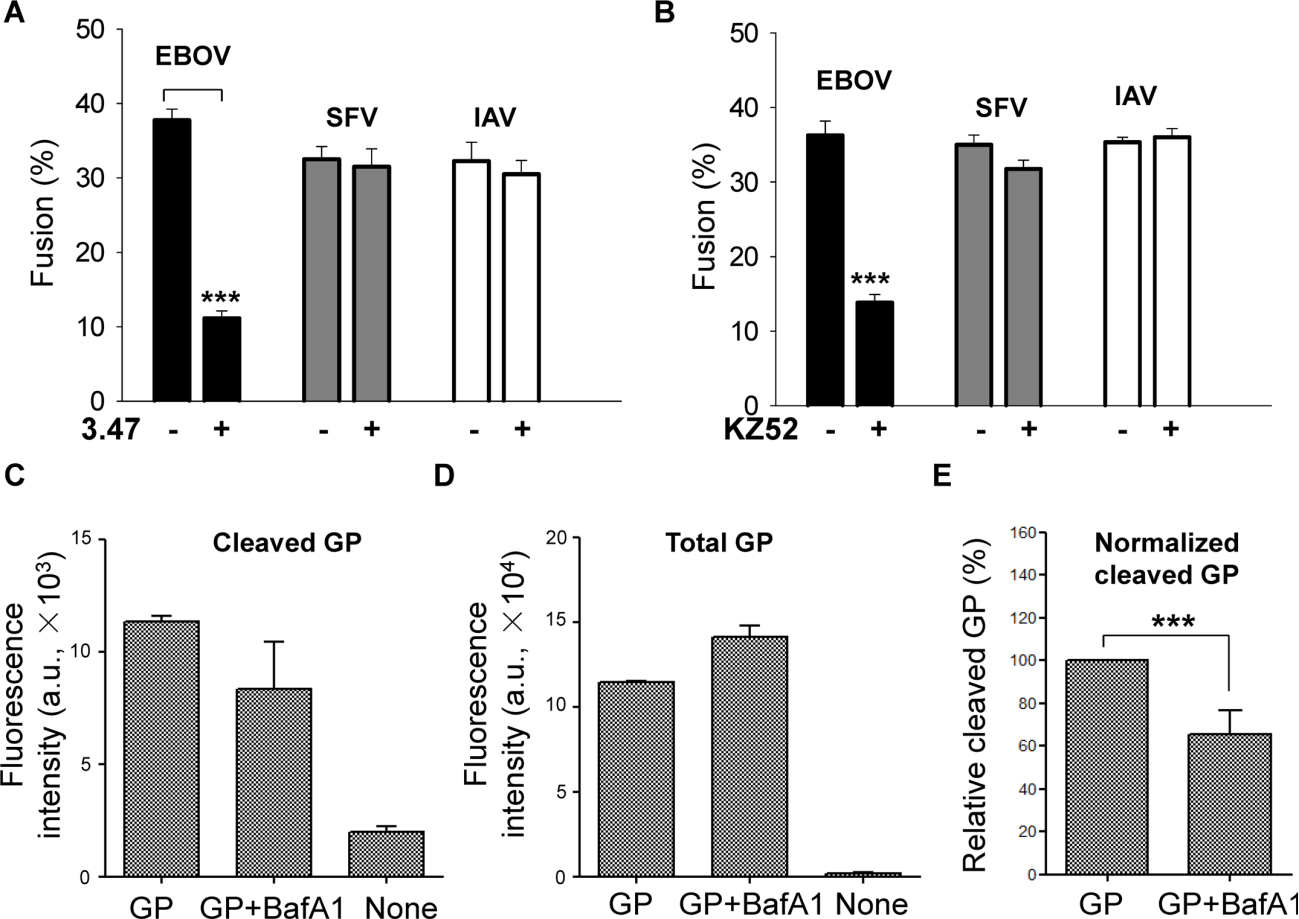
The small molecule inhibitor 3.47 and the neutralizing antibody KZ52 against EBOV GP blocked GP mediated fusion. **(A)** The inhibitor 3.47 (1 μM) was specific for EBOV GP, not affecting fusion mediated by SFV E1/E2 or IAV HA. **(B)** The inhibition of fusion by KZ52 (5.0 μg/ml) was also specific to EBOV GP. In all experiments of **(A)** and **(B)** a 10-min low pH pulse (pH 5.7 for EBOV GP, pH 5.4 for SFV E1-E2, pH 4.8 for IAV HA cleaved by trypsin) was employed. Results are at least four independent experiments. A 10-min pH 5.7 pulse augmented fusion for each protein. A comparison was made between pH 5.7 and 7.2 in each column. **(C)** Fluorescence intensity of the FITC-conjugated antibody measured by FACS showed that the presence of BafA1 reduced cleavage of plasma membrane GP. **(D)** The addition of BafA1 appeared to result in an increase of total GP in the plasma membrane. **(E)** BafA1 reduced the normalized cleaved GP on cell surface. In all figures, error bars are SEM; * p < 0.05; ** p < 0.01; *** p < 0.001.

We next tested 3.47, a small molecule inhibitor against NPC1, which prevents EBOV entry, as well as testing a neutralizing antibody (KZ52) against EBOV GP. We found that both significantly reduced EBOV GP-mediated cell-cell fusion (Fig 6A and 6B). The inhibitor 3.47 greatly reduced EBOV GP-mediated fusion but did not significantly alter cell-cell fusion induced by either Semliki Forest Virus (SFV) E1/E2 or IAV HA (Fig 6A, 3.47 at 1 μM). Similarly, the neutralizing antibody KZ52, which recognizes the interface between GP1 and GP2 [37], reduced EBOV GP-mediated fusion, but not SFV-E1/E2 or IAV HA-induced fusion (Fig 6B, KZ52 at 5 μg/ml). Higher concentrations of 3.47 completely inhibited fusion (S1A Fig), but fusion was not further reduced by increasing the concentration of KZ52 beyond that employed in Fig 6B (S1B Fig).

Another central fingerprint of GP-mediated fusion is inhibition of EBOV infectivity by Bafilomycin A1 (BafA1). By neutralizing endosomes, BafA1 inhibits infection, at least in part, by reducing cathepsin activity which in turn results is reduced cleavage of GP1. We found that addition of BafA1 (25 or 100 nM) reduced the amount of cleaved GP that appeared on the cell surface (Fig 6C, bar 2 vs bar 1). This occurred despite a consistently greater amount of total GP in the plasma membrane after the addition of BafA1 (Fig 6D). (This greater amount was unexpected. Possibly, BafA1 prevented lysosomal degradation of GP.) Normalizing the amount of cleaved GP by the total shows that cleavage of cell surface GP was significantly reduced by BafA1 (Fig 6E). Thus, all data support the conclusion that the aqueous dye spread we observe is due to fusion induced by EBOV GP.

### EBOV GP-mediated cell-cell fusion is maximal at mildly acidic pH

**Fig 7:**
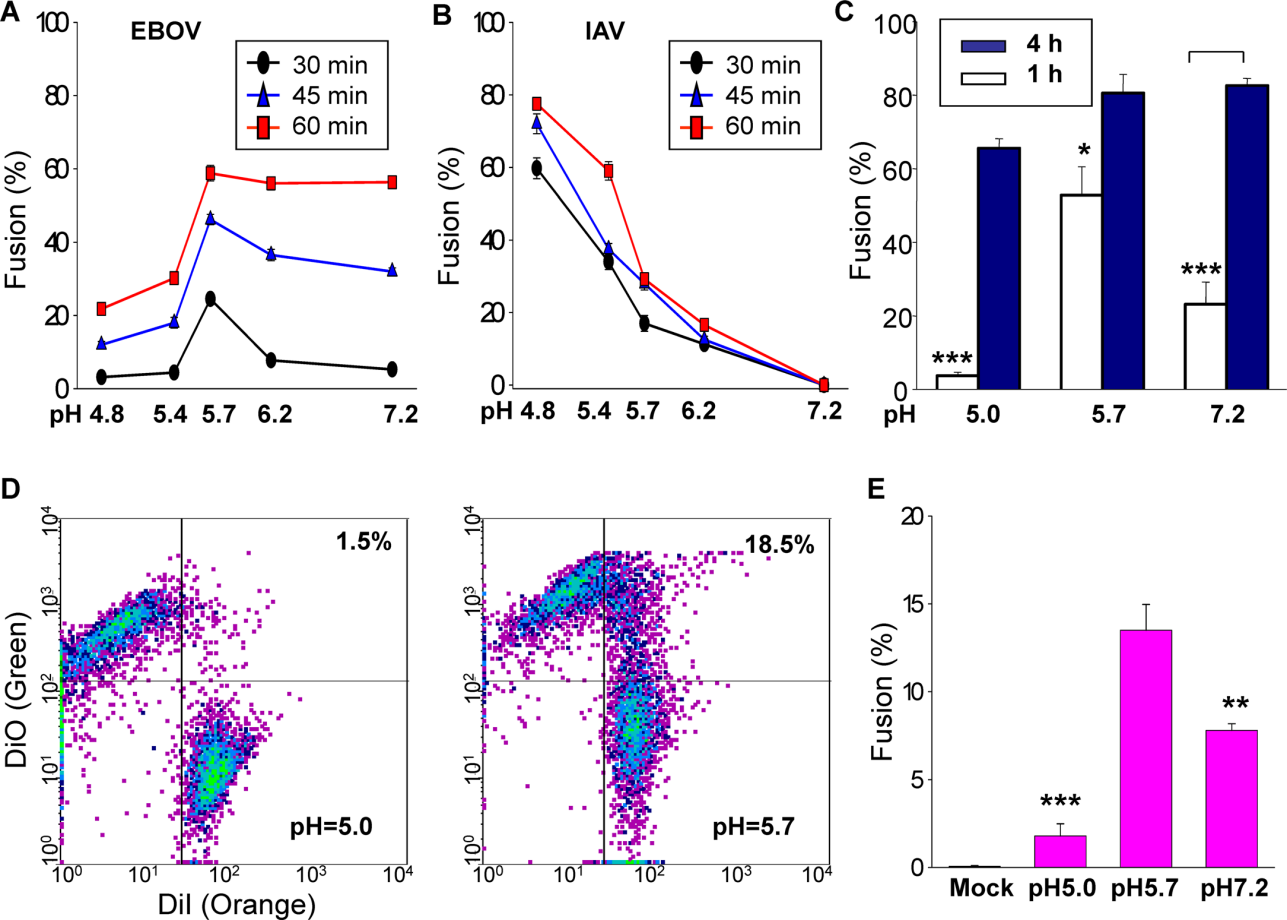
The extent of fusion mediated by EBOV GP is maximal at pH 5.7. **(A)** The extent of fusion between EBOV GP-expressing cells that were thermolysin-treated and target cells as a function of a 10-min, 37°C, low-pH pulse of the indicated pH values at varied times after reneutralization (30 min, black circle; 45 min, blue triangle; 1 h, red square). **(B)** The extent of cell-cell fusion induced by IAV HA is shown for the same conditions as in panel (A). Fusion progressively increased for lower pH pulses. **(C)** The extent of fusion as a function of pH (pH pulse applied at room temperature) after a 1 h (open bars) and 4 h (closed bars) reneutralization. **(D)** Effector cells were labeled with DiO and target cells with DiI. Both dyes were excited by a 488 nm laser; DiO emission was detected at 515 nm and DiI emission was recorded for 560 nm. For FACS measurements, trypsin and EDTA were added to cells prior to assaying; this treatment separates bound cells back into individual cells, but does not separate fused cells. Representative data for lipid dye mixing is shown for a 10-min pH 5.0 pulse (left panel), and a pH 5.7 pulse (right panel). **(E)** Average percentage of fusion as determined by lipid dye mixing as a function of pH (bars 2, 3, and 4). Lipid dye spread was negligible when using effector cells that were mock-transfected (bar 1). Thresholds for DiO and DiI were the same for all experiments and indicated on the two panels. Fusion was scored as the percentage of fluorescent particles above both thresholds (i.e., the third quadrant). Error bars are SEM (n = 6, for each bar) and extents of fusion were statistically compared to the extent for a pH 5.7 pulse. * p <0.05; *** p < 0.001.

Many of the effects of pH on kinetics and extents of EBOV GP-induced fusion we found were unexpected and quite different than those of pH-dependent fusion for other viral proteins. Notably, the extents of fusion did not monotonically increase as pH was progressively lowered, and the apparent pH dependence qualitatively varied with the times of reneutralization (Fig 4). After a pH 5.7 pulse, the extents of fusion were always greater than those achieved after more acidic pulses; following a pH 5.7 pulse (at short incubation times (i.e., 30 min) after the shift to neutral pH), more fusion was observed than for a less acidic pulse (Fig 7A). However, for pH pulses of 5.7 and above, as the reneutralization time was increased, the extents of fusion became less dependent on pH; fusion was independent of pH for 5.7 and above after a 1 h reneutralization (Fig 7A). In contrast, effector cells expressing IAV HA showed the typical and expected response of greater extents of fusion for lower pH values at all times after reneutralization; fusion events reached their full extents after a 30 min reneutralization (Fig 7B, using the same protocol as for EBOV GP experiments). Thus, IAV HA induces fusion more rapidly than does EBOV GP.

In separate experiments, we compared extents of EBOV GP-mediated fusion after a 4 h and 1 h reneutralization that followed 10 min, room temperature, acidic pH pulses (Fig 7C). After the 4 h reneutralization, fusion was relatively independent of the acidity of the pH pulse, and a low pH pulse did not greatly augment fusion (compare filled bars to open bars, Fig 7C). Equality in final extents of fusion at pH 5.7 and 7.2 could be a consequence of all cell pairs quickly fusing at low pH, thereby eliminating the possibility of further fusion, although we consider this unlikely.

In addition to single cell measurements of aqueous dye transfer, we also monitored lipid dye continuity between effector cells (treated with thermolysin) and target cells. We labeled effector cells with the lipophilic fluorescent dye DiO and labeled target cells with DiI and determined extents of fusion by flow cytometry (FACS). The double positive cells (i.e., the third quadrant) are clearly products of hemifusion or cell-cell fusion (Fig 7D). For effector cells treated with thermolysin, the percentage of fusion for the representative experiment was 18.5% at pH 5.7, the optimal pH for fusion (Fig 7D, second panel) and only 1.5% at pH 5.0 (first panel). Averaging six separate experiments for each condition, after a 2-h reneutralization, lipid mixing was greatest for a 10-min pH 5.7 pulse, and less for a pH 5.0 pulse than for cells maintained at neutral pH (Fig 7E). The approximately two-fold greater fusion determined by flow cytometry at pH 5.7 than at 7.2 is also in agreement with the data for spread of calcein (Fig 2). For mock-transfected effector cells, virtually no lipid dye spread was observed between effector and target cells (Fig 7E), in agreement with the aqueous dye spread measurements. Therefore it is clear that EBOV GP mediates a considerable amount of cell-cell fusion, and does so at an optimal pH of 5.7.

### EBOV GP-mediated fusion pores do not readily enlarge

**Fig 8:**
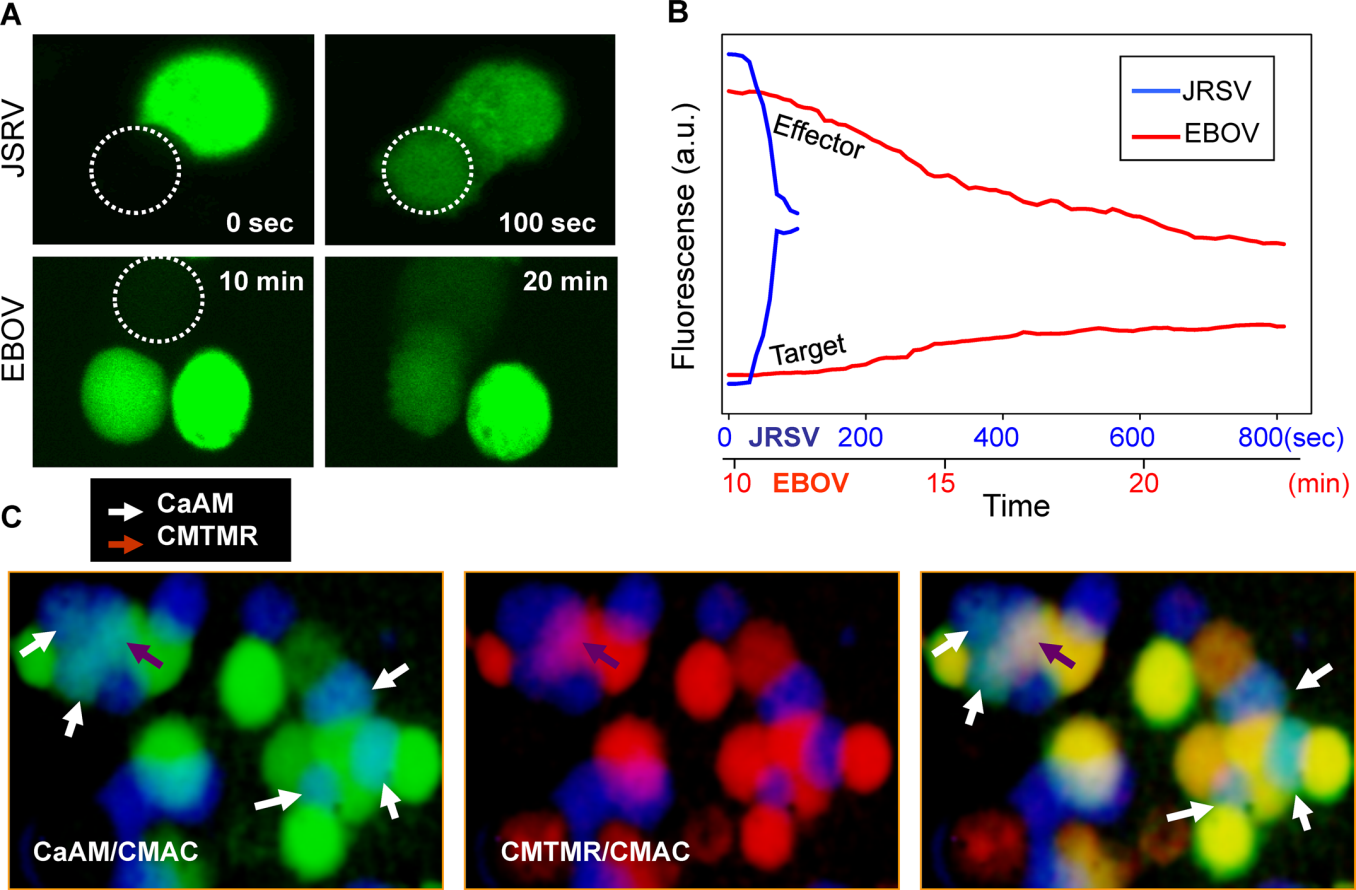
EBOV GP-induced fusion pores enlarge slowly. **(A)** The slow spread of calcein through EBOV GP-induced pores (upper panels) compared to JSRV Env-induced (lower panels) pores is shown. Calcein-AM was loaded into effector cells, and target cells were unlabeled. The dotted circle encloses the target cell receiving calcein. The moment that calcein first appears in the target cell is defined as time = 0. The right panels show calcein within the cells at t = 10 min. **(B)** A plot of calcein fluorescence in the effector and target cells as a function of time. The red line is the trace for an EBOV GP pore; the blue line is the trace for a JSRV Env pore. The upper time scale in units of hundreds of seconds refers to the JSRV Env pore; the lower time scale in units of minutes refers to the EBOV GP pore. The small difference in fluorescence of effector and target cells connected by the JSRV Env pore indicates that a small percentage of calcein is not free to transfer, possibly because it is bound to cellular elements. **(C)** Images of effector cells loaded with calcein AM (green) and CMTMR (red) and target cells loaded with CMAC (blue) taken after a 2 hr reneutralization at 37°C that followed a 10-min pH 5.7 pulse. Calcein has transferred (white arrows, left panel) from most of the effector cells in contact with target cells. CMTMR (middle panel) mixed with CMAC for only one cell (brown arrow). An overlay of calcein and CMTMR is shown in the third panel, with calcein (white arrows) and CMTMR (brown arrow) transfer shown.

Once calcein movement from effector to target cell commenced, it continued for EBOV GP-mediated fusion, but at an extremely slow rate. In general, the fluorescence due to calcein never equalized between target and effector cells for EBOV GP-induced fusion (Fig 8). In contrast, for fusion pores created by other viral fusion proteins [33,38], such as JSRV Env (Fig 8A, upper images), the fluorescence did equalize. It is possible that the EBOV GP pores eventually closed, preventing calcein from attaining the same concentration in effector and target cells. We therefore quantified the rate of transfer of calcein by plotting calcein fluorescence of effector and target cells as a function of time (Fig 8B). For EBOV GP-induced pores (red curve), the transfer occurred over a time course of tens of minutes, and over this period the increasing fluorescence of a target cell never equalized the decreasing fluorescence of an effector cell (Fig 8B). The fluorescence of the effector and target cells, on the other hand, equalized within a minute or so for JSRV Env-mediated pores (Fig 8B, blue curve). The exceedingly slow transfer of calcein is a further indicator that EBOV GP-mediated pores remained extremely small. The fact that calcein transferred, albeit slowly over long times, shows that the fusion pores did not irreversibly close (or if they did, new pores opened) within tens of minutes of formation. As a control, we added saponin to effector cells and measured release of calcein to be sure that the dye did not become compartmentalized and therefore failed to transfer for reasons unrelated to the size of the fusion pore. Release was fast from the saponin-treated cells and was almost complete within 10 s, demonstrating that the overwhelming majority of calcein was, in fact, free and mobile.

We further studied the size and rate of growth of EBOV GP-mediated fusion pores by assessing the size of dyes that can permeate these pores over time. We loaded effector cells with CMTMR in addition to calcein. CMTMR forms disulfide bonds with the tri-peptide glutathione, and these complexes are somewhat larger than calcein. The complexed glutathione can also form disulfide bonds with cytosolic proteins and hence CMTMR fluorescently labels proteins that are much larger than calcein. As a consequence, the size distribution of molecules labeled by CMTMR is expected to be quite diverse, some only somewhat larger than calcein and others very much larger. We found that CMTMR transferred for only 2–3% of the cell pairs for which calcein exchange occurred (Fig 8C). The relative inability of CMTMR to spread indicates that fusion pores typically did not enlarge sufficiently to allow passage of a molecule of the size of the nucleocapsid of EBOV. In actual viral infection, factors absent in our model system are probably promoting expansion of the fusion pore connecting an envelope and endosomal membrane.

**Fig 9:**
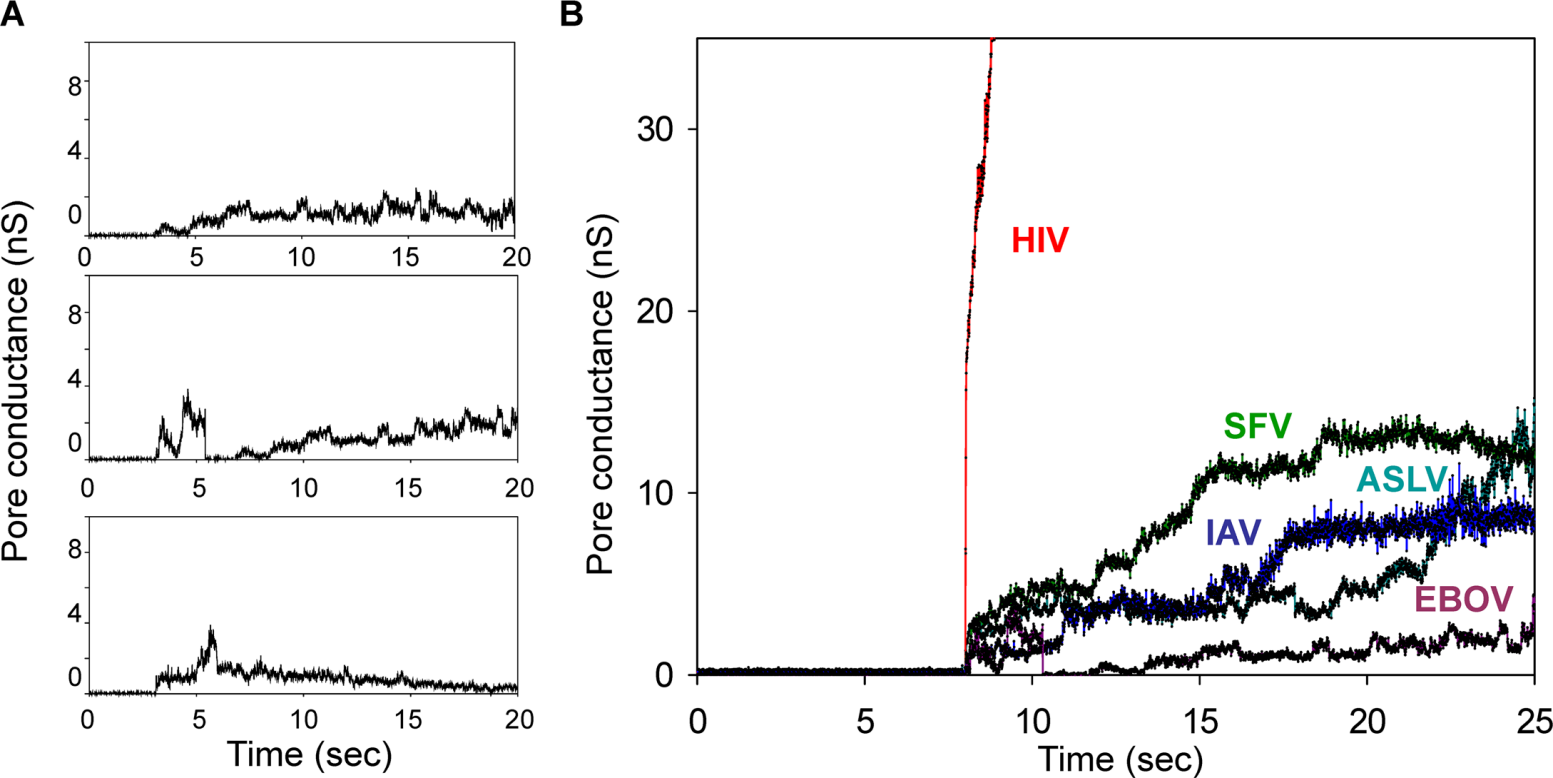
Electrical measurements demonstrate the slow, limited growth of EBOV GP-mediated pores. **(A)** Conductance traces of the three fusion pores, as detected by capacitance measurements are shown. None appreciably enlarged. **(B)** Representative pores induced by fusion proteins of different viruses are compared to the EBOV GP pore. HIV: Human Immunodefiency Virus 1; ASLV: Avian Sarcoma and Leukosis Virus. The illustrated representative EBOV GP pore is the same pore shown in the first trace of panel A.

We used electrical capacitance measurements to directly and quantitatively assess fusion pore size. The slow time course for EBOV GP-mediated fusion necessitated that the tight electrical seal between the patch pipette and plasma membrane be maintained for long times. This proved difficult in practice. We were able, however, to electrically observe pores between cell pairs in three cases, and in these cases the pores never enlarged within 30 s of formation and generally fluctuated within small values of conductance (Fig 9A). The conductance of the fluctuating pores did not return to baseline, showing that the pores did not close, but instead remained restricted to a small size. By way of comparison, it can be readily seen from representative traces of electrically measured fusion pores created by other viral proteins (Fig 9B) that fusion pores generally significantly enlarge over time. The absence of pore enlargement for EBOV GP suggests that many of the prior attempts at monitoring cell-cell fusion mediated by this fusion protein did not succeed because the reporter molecules that needed to permeate the fusion pore for detection of fusion were too large to pass through the pore. Although only three pores were electrophysiologically measured, the finding that each of them did not exhibit increased conductance over time implies that the slow passage of fluorescent dyes through them was not due to structures that prevent their access to the pores. Slow pore enlargement could be due to a number of factors, including slow recruitment and incorporation of additional copies of cleaved GP into the wall of the pore, or slow accumulation of lipids into the wall.

### Neutralization of intracellular compartments eliminates EBOV GP-mediated fusion

**Fig 10:**
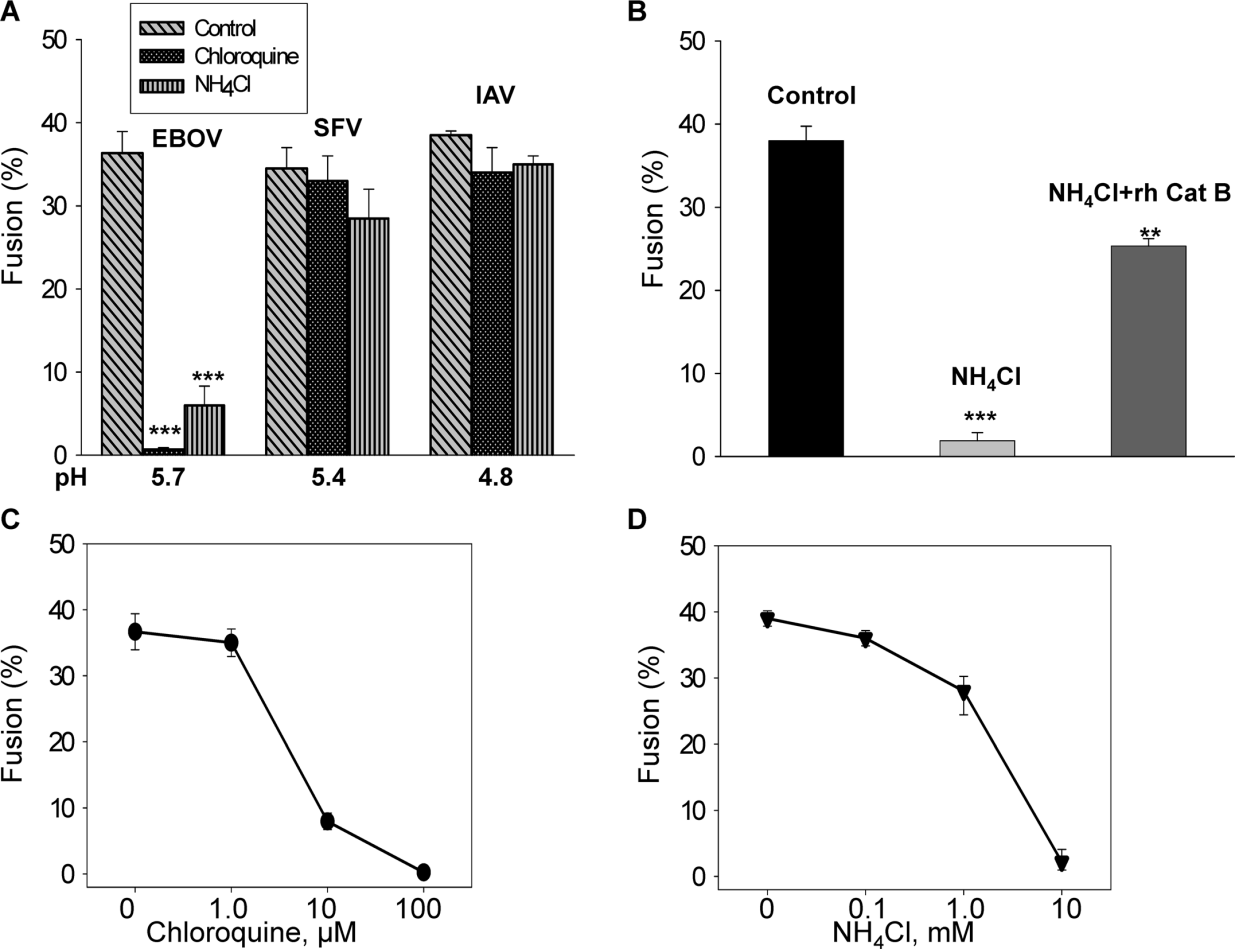
Neutralization of endosomes by ammonium chloride reduces fusion induced by EBOV GP. **(A)** Adding chloroquine (100 μM) or NH_4_Cl (10 mM) to the bathing solution greatly reduced EBOV GP-induced fusion after a pH 5.7 pulse (first set of three bars, endogenous cleavage (i.e., without thermolysin treatment) was employed. For fusion induced by SFV E1/E2 (with a pH 5.4 pulse, second set of three bars) or IAV HA (cleaved into HA1-HA2 subunits by a standard trypsin procedure, and employing a pH 4.8 pulse, third set of three bars), neither chloroquine nor NH_4_Cl greatly reduced fusion. (The addition of NH_4_Cl did, however, result in a slight decrease in SFV E1/E2-induced fusion.) Chloroquine or NH_4_Cl was added 20 min prior to acidification. Low pH was maintained for the standard 10 min, and fusion was measured 1 h after reneutralization. **(B)** The majority of the blockage of EBOV GP-induced fusion by NH_4_Cl was restored by the addition of recombinant cathepsins B (rh CatB) to the external solution immediately after a 10-min pH 5.7 pulse. The recombinant enzyme was present during the subsequent 1 hr reneutralization, with fusion then measured. **(C)** Dose-response curve for inhibition of fusion by chloroquine. **(D)** Dose-response curve for inhibition of fusion by NH_4_Cl. **(C)** and **(D)** are plotted semi-logarithmically

We added NH_4_Cl to external media to test whether acidic intracellular compartments were essential for EBOV GP-mediated cell-cell fusion. The addition of 10 mM NH_4_Cl greatly reduced fusion after a 10-min pH 5.7 pulse in the absence of thermolysin treatment, so as to avoid activating uncleaved EBOV GP on the cell surface (Fig 10A). In contrast, the addition of 10 mM NH_4_Cl did not affect fusion induced by an optimal pH pulse for either SFV E1/E2 or IAV HA (Fig 10A). Similarly, 100 μM chloroquine inhibited cell-cell fusion mediated by the fusion protein of EBOV, but not by the proteins from either SFV or IAV (Fig 10A). The elimination of fusion by the addition of 10 mM NH_4_Cl (bar 2; same conditions as in Fig 10A) was most likely caused by reducing cathepsin activity through neutralization of intracellular compartments: it was largely reversed by adding a recombinant cathepsin B to the external solution (Fig 10B, bar 3). Because the normally acidic intracellular compartments were neutralized by NH_4_Cl, the pool of EBOV GP on the cell surface that was previously uncleaved must have been cleaved by the added membrane-impermeant recombinant protease. The dose-response curves for inhibition of fusion by chloroquine (Fig 10C) or NH_4_Cl (Fig 10D) verified that inhibition of fusion is increased with increasing concentration of the neutralizing agent. Therefore, even if the external solution is acidified, EBOV GP-mediated cell-cell fusion does not occur unless the acidity of intracellular organelles is maintained. We conclude that EBOV GP present on the cell surface requires an intracellular compartment for cleavage, as is consistent with previous reports. It is possible, however, that there are copies of cathepsins in the plasma membrane, and acidification of the external solution activates them to cleave EBOV GP.

### EBOV GP cycling between plasma and intracellular membranes affects cell-cell fusion

Proteinase K (PK) has proved useful for assessing conformational changes that viral proteins undergo at different stages of fusion [30,39]. We found that EBOV GP was PK-sensitive for all steps of the fusion process (S1 Text and S2A Fig), that fusion was restored over time after removing PK (S2B Fig), and that normal cellular trafficking of protein led to replacement of proteolytically digested GP with newly delivered intact GP (S3 Fig).

We also used Brefeldin A (BFA, 50 μM)—an inhibitor of trafficking from endoplasmic reticulum to Golgi—to further characterize the consequences for fusion of altering intracellular trafficking of EBOV GP. Here we found that treatments expected to increase the amounts of cleaved EBOV GP on the cell surface led to greater extents of fusion (S1 Text and S4 Fig).

### pH-dependent cathepsin activity is essential for GP-mediated fusion

**Fig 11:**
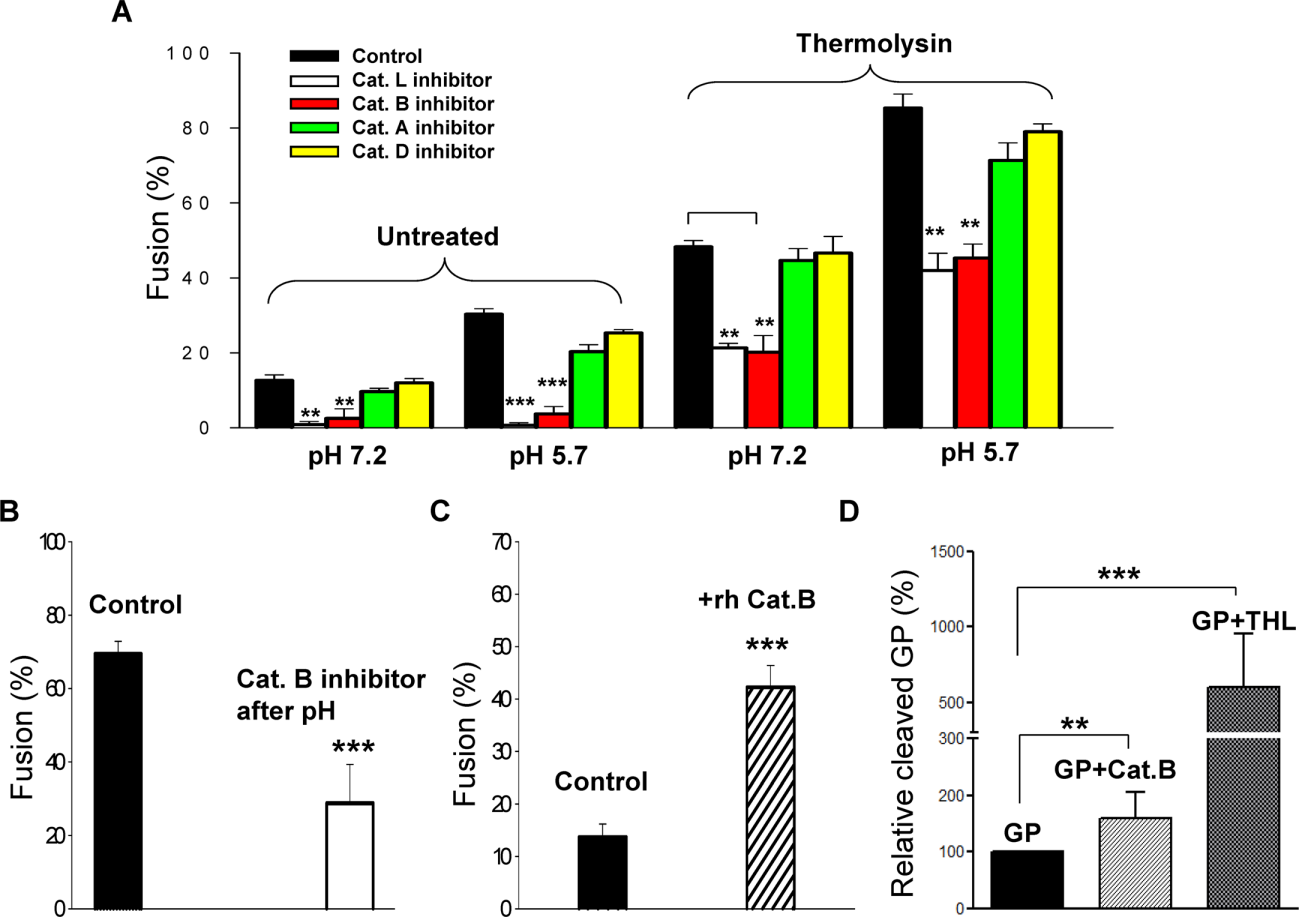
Blocking cathepsins that cleave EBOV GP reduces fusion. **(A)** An inhibitor of cathepsin L (second bar of each set of bars), cathepsin B (third bar of each set of bars), cathepsin A (fourth bar of each set) and cathepsin D (fifth bar of each set) are shown and compared to the case in which the inhibitor was not added (first bar of each set, control). For effector cells not thermolysin-treated, fusion experiments were performed in the absence of a low-pH pulse (first set of columns) and with a pH 5.7 pulse (second set of columns). For cells treated with thermolysin, cathepsin inhibitors were added prior to the thermolysin treatment and maintained throughout the experiments. For thermolysin-treated cells, fusion was measured for the case without (third set of bars) and with a low pH pulse (fourth set). Only inhibitors of cathepsin L or B diminished fusion, and they did so for all conditions. Each of the four cathepsin inhibitors was added (separately) at the time of mixing effector and target cells. The cathepsin L and cathepsin B inhibitors reduced fusion to the same extent at pH 5.7 and pH 7.2. The cathepsin B and D inhibitors were without effect. Fusion was measured 2 hr after the low pH pulse. The concentration of all inhibitors was 100 μM, a high concentration to ensure maximal inhibition. **(B)** In contrast to the experiments in (A), the cathepsin B inhibitor was added subsequent to the low pH 5.7 pulse (second bar). Less fusion occurred than for control (filled bar). **(C)** A recombinant human cathepsin B (rh CatB, 200 μM) was added to effector cells that had not been treated with thermolysin. The addition of rh CatB (hashed bar) led to substantially increased fusion (filled bar, control). A 10-min pH 5.7 pulse was applied to promote fusion. The extents of fusion were measured after a 2-hr reneutralization for all experiments of this figure. Error bars are SEM. **(D)** 293T cells were co-transfected with plasmids encoding EBOV GP and cathepsin B. Cleaved GP on the plasma membrane was measured by flow cytometry using sNPC1, and the cleaved form was normalized by the total GP (measured by anti-FLAG), as described in Fig 4. Alternatively, GP-expressing cells were treated with thermolysin, and cleaved GP was measured and normalized as described. **p<0.01; ***p<0.001.

For virus internalized in endosomes, EBOV GP is believed to be cleaved by cathepsins B and L, but not by cathepsins A or D. We prevented cathepsin-induced cleavage by treating bound effector and target cells with a cathepsin B inhibitor (CA-074) or a cathepsin L inhibitor (Z-FY-CHO). In the absence of thermolysin treatment, the inhibitors led to significantly reduced fusion at both neutral and low pH (Fig 11A, compare “untreated” and “treated”: as always, changes of solutions containing membrane-impermeant buffers were used to control pH). Using inhibitors against cathepsin A (lactacystin) or cathepsin D (pepstatin A)–neither of which is thought to cleave EBOV GP–did not lead to reduced fusion using the same protocol as for the cathepsin B and L inhibition experiments (Fig 11A). These results provide strong support that fusion observed in our experiments in the absence of thermolysin treatment is due to, at least in part, copies of EBOV GP on the cell surface that have their GP1 subunits cleaved by cathepsins. These results also document that neither cathepsin A or D cleaves EBOV into a fusion-competent form. From the results as a whole, it is clear that low pH does not induce fusion unless the GP1 subunit has been cleaved. It is known that cathepsin activity is increased by acidity. We suggest that low pH acts, at least in part, by augmenting cathepsin activity on the cell surface. The same pattern of pH-dependence of fusion was observed for effector cells treated with thermolysin while cathepsin activity was continually inhibited: fusion was dependent on pH and was significantly reduced by the cathepsin B inhibitor or the cathepsin L inhibitor (Fig 11A, thermolysin-treated, bars 2 and 3 of each set of columns), but was relatively unaffected by cathepsin A or D inhibitors (bars 4 and 5). Cell-cell fusion exhibits a maximum at pH 5.7 (column 4 compared to column 1–3). Several cathepsins exhibit maximal activity in the pH range of 5.5 to 6.8 [40], so the maximum extent of fusion at pH 5.7 would likely be due to the pH dependence of cathepsin activity on the cell surface.

**Fig 12:**
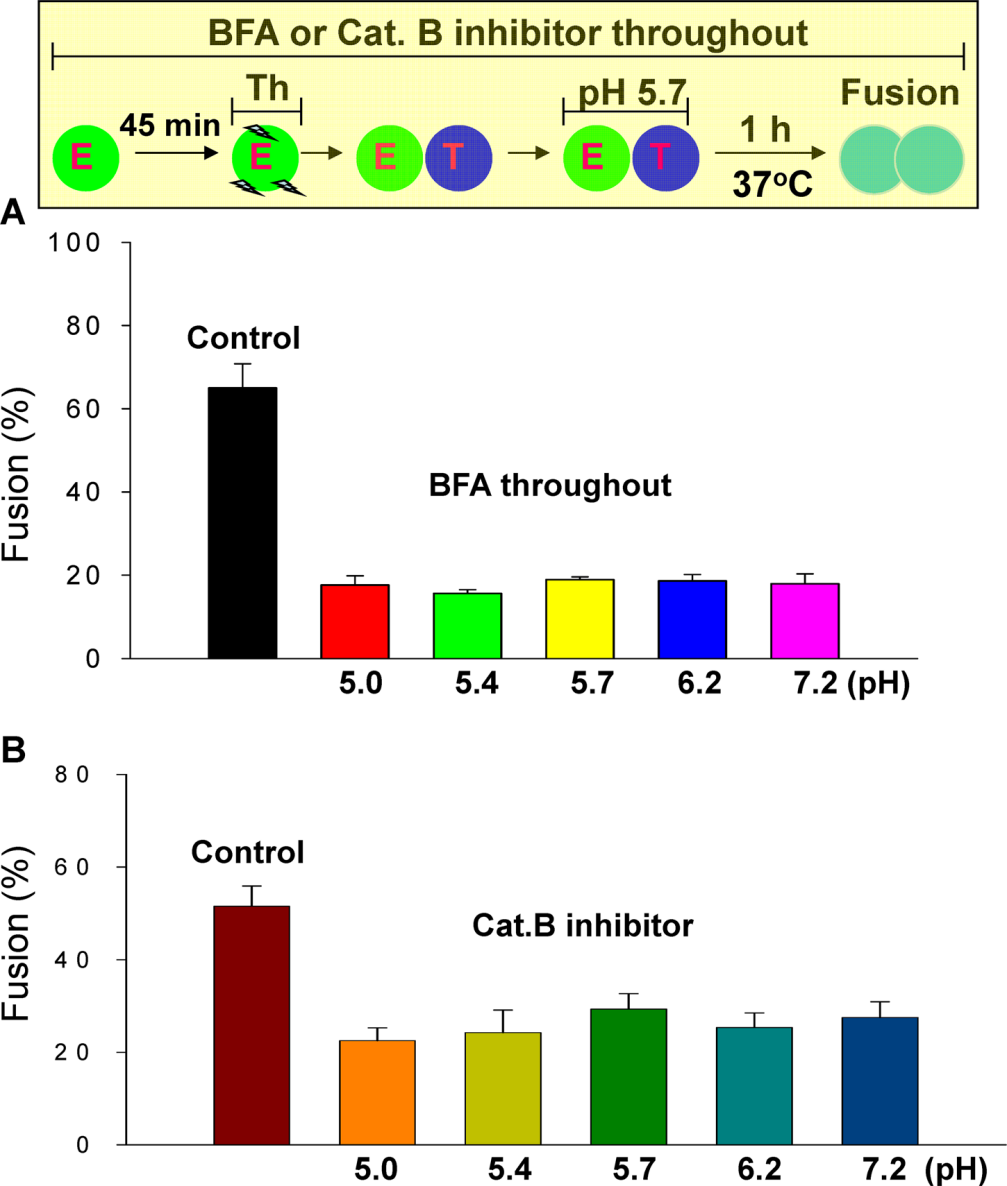
After EBOV GP is cleaved, GP-mediated fusion is independent of pH. **(A)** Blocking delivery of EBOV GP to the cell surface yields pH-independent fusion: BFA was added to effector cells 45 min prior to mixing them with target cells, and then maintained to prevent EBOV GP trafficking to the plasma membrane. This eliminated the pH-dependence of fusion for thermolysin-treated effector cells. **(B)** Utilizing the standard fusion protocol, but with a cathepsin B inhibitor (100 μM) used to pretreat effector cells for 45 min and maintained at all times, fusion of thermolysin-treated effector cells with target cells was independent of pH. Fusion was much greater in the absence of the inhibitor. More importantly, inhibition of cathepsin activity eliminates the pH-dependence of fusion.

Control experiments provide additional support for the conclusion that cathepsins aid EBOV GP-mediated fusion between cells. Blocking cathepsin B (by adding the cathepsin B inhibitor) immediately after application of an acidic pH pulse resulted in a substantial reduction in the extent of fusion after a 2-h reneutralization (Fig 11B, effector cells were thermolysin-treated). The reduction from the control was ~2-fold; a 2-fold reduction also occurred when the cathepsin B inhibitor was constantly present (see Fig 12B). The nearly equal percentages of inhibition of fusion are expected, since in the presence of the cathepsin inhibitor, uncleaved copies of EBOV GP would not be cleaved during the period of reneutralization. Thus, low pH appears to promote cleavage of EBOV GP by cathepsins on the cell surface. Incubating effector cells that were not treated with thermolysin with a recombinant human cathepsin B (rhCat B) (Fig 11C, bar 2) increased fusion significantly over the control (bar 1). The simplest explanation for this increase is that the recombinant protein led to a higher level of GP1 cleavage than that induced by endogenous cellular cathepsins. To explicitly test whether increasing the activity of cathepsin increased the likelihood that GP on the cell surface was cleaved, we cotransfected cells to express GP and cathepsin B and used sNPC1 to measure the percentage of GP in the plasma membrane that was cleaved (as described for Fig 4). This percentage was greater (Fig 11, column 2) than the control (column 1) in the presence of cathepsin B transfection. Using the same techniques, we also showed that adding thermolysin to solution did indeed increase cleavage of cell surface GP (Fig 11D).

### Altering EBOV GP cycling and inhibiting cathepsin activity show that once GP is cleaved, fusion is independent of pH

Does low pH directly cause conformational changes in EBOV GP to induce fusion, or does it work via increasing the activity of cathepsins, or both [7,20]? We were able to approach these questions by using the ability of BFA to effectively block delivery of EBOV GP to the cell surface and, independently, by using cathepsin inhibitors to prevent GP cleavage. We incubated effector cells with BFA for 45 min to prevent further delivery of EBOV GP to the plasma membrane prior to a thermolysin-treatment, and maintained the presence of the drug during all solution changes. The extent of fusion was independent of pH, and considerably less than when the trafficking inhibitor was not employed (Fig 12A). The clear conclusion is that, with BFA present, all fusion was caused by copies of EBOV GP that had been cleaved by thermolysin and that remained on the cell surface. The finding that pH pulses did not affect the extent of fusion at all shows that acidity did not promote the conformational changes in cleaved EBOV GP that would lead to fusion.

We inhibited cathepsin activity to further test the conclusion that once cleaved, EBOV GP no longer requires low pH to induce fusion. We performed experiments in which CA-074, a cathepsin B inhibitor, was continually present (Fig 12B). The inhibitor was added to isolated effector cells and maintained for 45 min to ensure that all EBOV GP delivered to the plasma membrane was not cleaved. Effector cells were then thermolysin-treated, always maintaining the inhibitor. These cells were bound to target cells, and the external solution was acidified to pH 5.7; after reneutralization, cells were maintained for 1 h at 37°C, with all manipulations performed in the presence of the inhibitor (Fig 12B, experimental protocol illustrated on top). The extent of fusion was greater when the inhibitor was not added (control): this indicates that delivery to the cell surface of EBOV GP cleaved by endosomal cathepsin (subsequent to thermolysin treatment) significantly contributes to fusion. More importantly—and central to the mechanism of EBOV GP-mediated fusion—the extent of fusion was independent of pH. This finding strongly implies that acidic conditions have no direct effect on EBOV GP-mediated fusion. The pH dependence of fusion is solely due to the ability of cathepsin to cleave EBOV GP; once cleaved, acidic conditions directly induce conformational changes in cleaved EBOV GP that lead to fusion.

## Discussion

Using both cytoplasmic and membrane dye transfer assays, we established that known properties of EBOV fusion occurring within endosomes are replicated by our cell-cell fusion system and that specific inhibitors of EBOV infection—the small molecule inhibitor 3.47 and a neutralizing antibody KZ52—block fusion. The inhibition of EBOV GP-mediated cell-cell fusion (but not IAV HA or SFV E1/E2 fusion) by the lysosomotropic agents NH_4_Cl and chloroquine is expected: EBOV GP cleavage is eliminated because cathepsin activity is greatly reduced by neutralization of endosomes; inhibiting cathepsin activity reduces cleavage of EBOV GP [41,42]. We also showed that copies of NPC1 reside in the target membrane and some GP resides in the plasma membrane, and that a fraction of the GP is properly cleaved. It is virtually certain that the cell-cell fusion process investigated in the present study is mediated by EBOV GP.

It is likely that past lack of success in observing cell-cell fusion is attributable to the fact that the fusion pore mediated by EBOV GP on the cell surface remains small. Over the time scales of electrical measurements, the pore does not enlarge at all. Based on fluorescence dye spread measurements, it enlarges more slowly and to a lesser extent than any other pore mediated by a viral fusion protein of which we are aware, and it may even tend to close. The EBOV GP fusion pore is large enough to allow the passage of calcein, but just barely. There is little doubt that the fluorescent dye CMTMR does not permeate the pore because virtually all of it complexes with proteins; the complex becomes permeable only after a pore enlarges. Electrical measurements directly demonstrate that the EBOV GP-induced pore remains small. Over the course of time in our cell-cell fusion experiments, EBOV GP-mediated fusion pores do not significantly enlarge.

The question now becomes: how readily does a fusion pore enlarge when connecting an EBOV envelope with an endosomal membrane? This fusion pore must expand to sizes that permit passage of the large viral nucleocapsid. Four major possibilities present themselves: (i) the necessary enlargement is extremely slow for the endosome-viral pores; (ii) elements engaging plasma but not endosomal membranes, such as cytoskeleton, retard the growth of fusion pores; (iii) a protein (such as the two-pore calcium channel, present in the endosomal compartments that support EBOV fusion [43]) is required for pore enlargement; (iv) control of calcium concentrations (e.g., through the two-pore channels) regulates fusion pore formation or enlargement in endosomes. Methods to monitor the formation and growth of fusion pores of EBOV GP-bearing viral particles within endosomes will be needed to answer these questions [44].

We have unambiguously shown that a fraction of EBOV GP on the cell surface is cleaved. By using the GP^furin^ construct we also demonstrated that increased cleavage correlates with greater fusion. In addition, we functionally evaluated the cleavage status of EBOV GP on the cell surface by adding a water-soluble recombinant cathepsin B or thermolysin to solution and found that these proteases promoted fusion. Late endosomes and lysosomes are generally thought to be the cellular site of cleavage of EBOV GP by cathepsins [45]; it is likely that a fraction of EBOV GP is cleaved within endosomes and then recycled to the plasma membrane where it mediates cell-cell fusion, independent of pH (Fig 12). We suggest that uncleaved EBOV GP that reaches the surface is cleaved upon acidification of the external solution by cathepsins within the plasma membrane. Thermolysin cleaves uncleaved copies of EBOV GP that are delivered to the cell surface, accounting for the enhancement of fusion by the addition of the protease. Regardless of the site of GP cleavage, an appreciable fraction of the GP1 subunit is indeed cleaved into its fusion-competent form after the addition of thermolysin.

NPC1 serves as receptor for EBOV GP in endosomes, and is essential for the virus to infect a cell. We have now shown that NPC1 is not confined only to intracellular membranes, but rather that some copies reside in plasma membranes. Our finding that sNPC1 promotes EBOV GP-mediated cell-cell fusion suggests that domain C of NPC1 alone is sufficient to induce the needed conformational changes in the fusion protein.

It is well established that the extents of cell-cell fusion correlate with the levels of viral fusion protein expression on cell surfaces [46,47]. Thus, it is not surprising that the extents of cell-cell fusion induced by EBOV GP are affected by its delivery to, and loss from, plasma membranes. For some viral fusion proteins, such as the paramyxovirus Hendra and Nipah virus F proteins, and SARS coronavirus S protein, cell-cell fusion is sensitive to protein cycling [48,49,50]. These proteins require acidic intracellular compartments for cleavage: endosomes for Nipah virus [51], and endosomes and the Trans-Golgi Network for Hendra virus [52]. But the dependence of cell-cell fusion on protein trafficking is unusual for typical pH-dependent viral fusion proteins; for these proteins acidity does not promote cleavage, but instead directly induces conformational changes [53]. Once activated, these typical low-pH-dependent fusion proteins quickly inactivate if they do not promote fusion [54]. Hence, protein delivered to the cell surface subsequent to an acidic pulse will not be able to promote fusion. In contrast, proteins that induce fusion at neutral pH will promote fusion once they are delivered to the cell surface. Our results show that EBOV GP cleaved by endosomal cathepsins are no longer sensitive to pH and therefore can induce fusion once they arrive at the plasma membrane.

Infectivity is subject to processes other than fusion, and so infectivity need not always correlate with extents of cell-cell fusion. For example, it has recently been shown that the activity of two-pore calcium channels in endosomes is required for EBOV infection [43], and that EBOV infects by fusing to endosomal membranes that contain both NPC1 and the two-pore channel [44]. Tetrandrine blocks these channels and inhibits EBOV infection. We found that tetrandrine (150 nM) did not affect EBOV GP-mediated cell-cell fusion.

It is notable that the extent of fusion that occurs after 4 h at neutral pH was roughly equal to the extent that follows a pH 5.7 pulse. Because fusion induced by cleaved GP is pH-independent, we interpret the continual increase in fusion at neutral pH over time to be a consequence of intracellular trafficking: new copies of EBOV GP continually replace or supplement old copies and these new/supplemented, cleaved copies can cause fusion between cells that had not previously fused. Thus, acidification likely promotes more fusion at early times through activation of surface cathepsins that cleave EBOV GP. However, it is not presently clear why fusion kinetics is faster after a pH 5.7 pulse than after a pH 5.4 (or more acidic) pulse. The pH dependence of cathepsin activity is complicated [55]. While activity generally increases with acidification, some cathepsins exhibit the same activity in the range of pH 7 as at lower values of pH [56]. For others, activities are maximal at an intermediate pH, such as 5.7 [40]. Also, the pH dependence of cathepsin activity varies with environment and conditions, such as redox potentials on each side of the membrane in which a cathepsin resides [57,58]. Any relevant cathepsins (e.g., B or L) on the cell surface can, at their optimal pH, cleave EBOV GP. A direct test of whether EBOV GP on the cell surface is maximally cleaved by cathepsins at pH 5.7 will require methods to measure the percentage of EBOV GP that is cleaved as well as cathepsin activity at the cell surface.

The role of acidic pH in EBOV fusion has been debated in the field [7,20,59]. Our data unambiguously show that cell-cell fusion is regulated by extracellular pH. The acidity of the extracellular solution can, in principle, augment both the activity of cathepsins that reside in the plasma membrane and directly promote conformational changes of cleaved EBOV GP on the cell surface. (Although cathepsins are regarded as endosomal membrane proteins, some copies also likely reside in plasma membranes from which many endosomes derive [60]). The great reductions in fusion caused by inhibition of cathepsin activity and recovery of fusion by addition of a recombinant cathepsin establish that cathepsins’ activity at the cell surface is consequential. Independent experiments in which cathepsin activity was inhibited or delivery of protein to cell surface was blocked show that the pH-dependence of fusion is eliminated once EBOV GP is cleaved. This demonstrates that fusion mediated by the cleaved form is intrinsically pH-independent. That is, cleaved EBOV GP is essentially a neutral pH fusion-inducing protein; all the experimentally observed and biological relevant pH-dependence is a consequence of cathepsin activity. The faster kinetics of cell-cell fusion after a pH 5.7 pulse than for pulses at higher values can be accounted for by greater cleavage of EBOV at the cells surface at pH 5.7. A previous study used model peptides to mimic the six-helix bundle of EBOV GP2 and found that low pH increased bundle stability [61]. The stage of fusion in which bundle formation occurs has not been identified for EBOV GP. It may be, for example, that the bundles form subsequent to pore formation, as occurs for HIV Env [29], and that increased bundle stability aids pore enlargement, but not fusion itself [62]. Alternatively, the model peptide may not mimic bundle stability within a full length, structurally intact GP.

As a general rule, fusion kinetics for viral proteins that induce fusion at neutral pH are slower than for proteins that utilize low pH as a trigger. This could explain the slow fusion kinetics of EBOV GP mediated fusion, despite classification as a low pH-requiring process. Alternatively, a need to continually deliver EBOV GP could be the reason EBOV GP-mediated cell-cell fusion is slow. The development of an experimentally convenient system of EBOV GP-mediated fusion should make it possible to determine molecular mechanisms by which EBOV releases its genome into infected cells.

**Fig 13:**
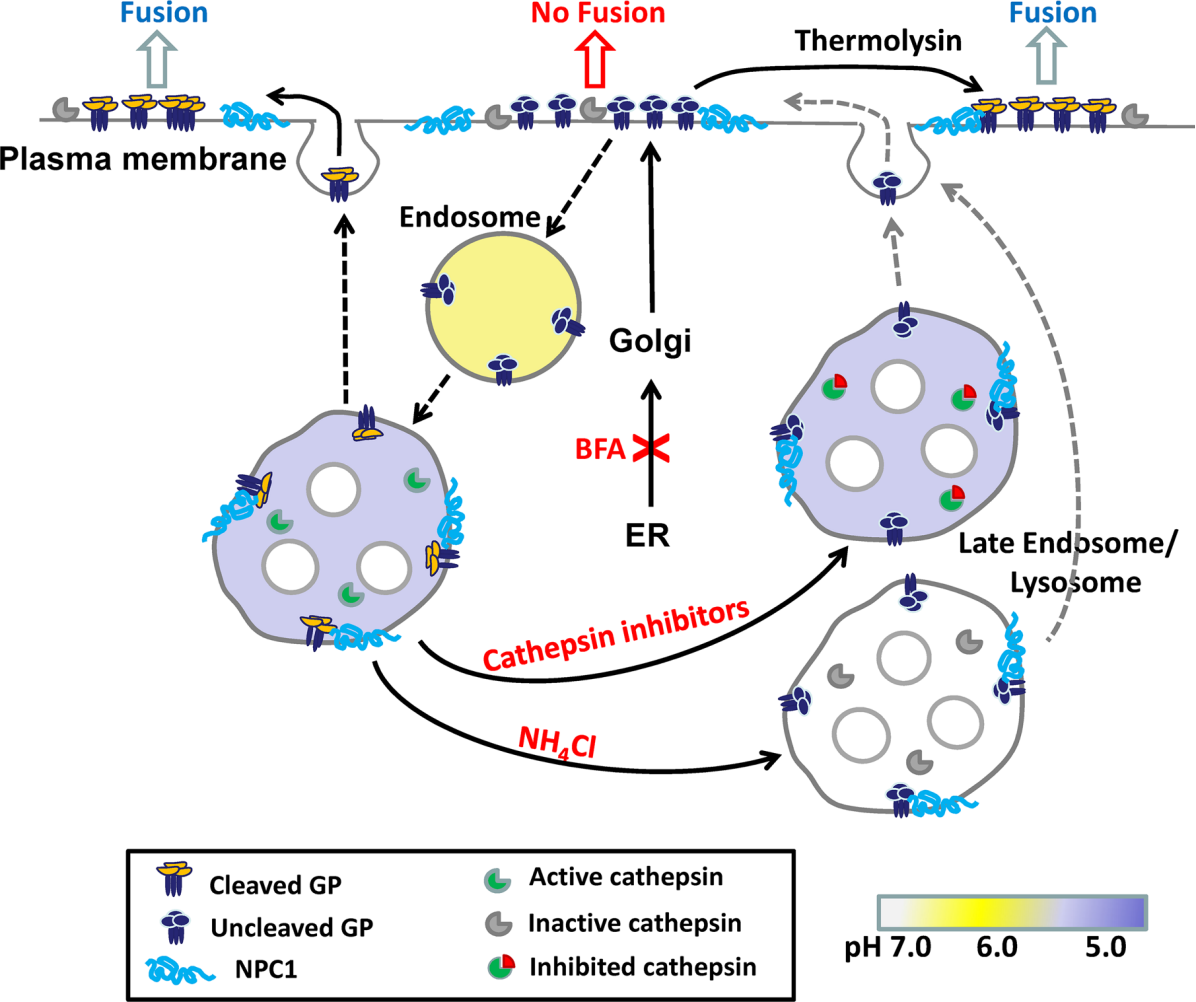
Schematic diagram illustrating control of fusion by cleavage of EBOV GP and its transport to the cell surface. EBOV GP is synthesized in ER, transported to Golgi complexes where it is processed into GP1 and GP2 subunits, and ultimately targeted to the plasma membrane. EBOV GP can undergo endocytosis from the plasma membrane and eventually reach late endosomes and lysosomes. It is then further cleaved by cellular cathepsins, bound by NPC1, and recycled back to plasma membrane. Alternatively, EBOV GP is directly cleaved by cathepsins on the plasma membrane or by thermolysin treatment. EBOV GP proteins do not permanently remain on the surface, but rather undergo continual delivery and removal. Cleaved GP accumulates at potential fusion sites, leading to the observed increased fusion over time. Solid arrows denote pathways definitively established in the present study. Dashed arrows denote pathways that are likely to occur based on data of the present study. Light dashed arrows denote pathways suggested by data of the present study.

Our results and conclusions are diagrammatically summarized in Fig 13. NPC1 is an intracellular receptor for EBOV GP within endosomes. But, as we have shown, NPC1 can also reside in the plasma membrane. Endosomal cathepsins cleave EBOV GP, and any cathepsins that reside in plasma membranes will also cleave surface GP upon acidification of the external solution. Both cleaved and uncleaved copies of EBOV GP are continually delivered to and retrieved from the surface, and hence intracellular trafficking contributes to extents and kinetics of fusion. But binding of EBOV GP to the target membrane should inhibit endocytotic retrieval. Consequently, EBOV GP (both cleaved and uncleaved) should accumulate at potential fusion sites, leading to more fusion over time. Preventing acidification of endosomes to block cleavage of EBOV GP, or inhibiting delivery of the protein to the cell surface, greatly reduces fusion. Acidification of the external solution to pH 5.7 increases the activity of cathepsins that reside in the cell surface, and this results in additional cleavage of EBOV GP. The addition of thermolysin converts all surface GP to the cleaved form, thereby resulting in the maximal extent of fusion. That cell-cell fusion induced by cleaved EBOV GP does not depend on pH, provides critical insight into the mechanism of EBOV entry and infection.

## Materials and Methods

### Reagents and constructs

Purchased reagents were: Lactacystin (a cathepsin A inhibitor, Santa Cruz Biotechnology, Dallas, TX); pepstatin A (a cathpesin D inhibitor, Santa Cruz Biotechnology); Cathepsin L inhibitor (catalog no. sc-3132, Santa Cruz Biotechnology); CA-074 (Cathespin B inhibitor, Calbiochem); Recombinant human cathepsin B (R &D Systems, Fisher Scientific), Brefeldin A (Cayman Chemicals, Ann Arbor, MI),); poly-lysine (M.W. 70 kD, Sigma); bovine serum albumin (BSA, Sigma), Thermolysin (Sigma); Proteinase K, chlorpromazine (CPZ) (Sigma), lentiviral shRNA targeting NPC1 (Sigma), anti-NPC1 (LifeSpan BioSciences, Seattle, WA), anti-FLAG and anti-ß-actin antibody (Sigma); anti-GP1 anitbody (gift of James Cunningham, Harvard Medical School, Boston, MA). PBS^++^ and DMEM were obtained from Gibco (Grand Island, NY). All fluorescent probes were purchased from Molecular Probes (Life Technologies, Eugene, OR). The mucin-deleted EBOV GP construct was originally obtained from David Sanders (Purdue University, West Lafayette, IN). For this work, we mainly used an N-terminal FLAG-tagged, mucin-deleted EBOV GP construct by replacing the signal peptide of GP with that of preprotrypsin followed by a FLAG sequence. All GP mutants, including GP^furin^, were made by overlapping PCR using the FLAG-tagged GP construct as the template. The plasmid to express JSRV Env has been described [38]. To express IAV HA for dye spread experiments, we used the X31 strain (plasmid provided by Judith White, University of Virginia, Charlottesville, VA). A standard calcium phosphate method was used to express SFV E1/E2 via transfection of the pCB3-wt vector, plasmid provided by Margaret Kielian, Albert Einstein College of Medicine, Bronx, NY [63]. A small molecular inhibitor, 3.47, was a gift of James Cunningham (Harvard Medical School, Boston, MA).

### Cell lines

HEK 293T and COS7 cells employed have been previously described [64]. The HEK 293T cells stably expressing were generated by transducing cells with pBabe retroviral vector expressing NPC1 (gift of Kartik Chandran) followed by puromycin selection (Sigma, 2 μg/ml). All cells were grown in Dulbecco’s modified Eagle’s (DMEM) medium, supplemented with 0.5% penicillin/streptomycin plus 10% fetal bovine serum (FBS).

### Fusion experiments using aqueous dye transfer

For all experiments using EBOV GP, COS7 cells were maintained in Eagle’s Medium with glucose, L-glutamine, and sodium pyruvate, supplemented with 10% Cosmic Calf Serum (HyClone, Logan, Utah), Pen Strep (Gibco), and 0.5 mg/ml G418 Sulfate (Cell Gro, Manassas, VA), and transfected to express EBOV GP by a standard calcium phosphate procedure [65]. About ~ 2x10^6^ cells were loaded with 1.5 μM calcein-AM as previously described [29] and sometimes coloaded with 1 μM 5-(and-6)-(((4-chloromethyl)benzoyl)amino)tetramethylrhodamine) (CMTMR) [29]. If these effector cells were thermolysin-treated to cleave EBOV GP, 200 μg/ml thermolysin was incubated with the cells for 20 min at room temperature. Exchanging the solution with DMEM removed thermolysin; residual thermolysin was further removed by spinning down the cells three times and replacing the aqueous solution. HEK 293T cells were maintained in the same media and in the same way as COS7 cells and were used as targets. ~ 2x10^6^ cells were loaded with 20 μM CMAC. Effector cells were mixed, including a gentle vortex—in a tube containing either PBS^++^ (sometimes supplemented with 1 mg/ml BSA) or DMEM—with the labeled target HEK 293T cells. The cells were added into polylysine-coated (1 mg/ml) wells of an 8-well slide (Thermo Fisher) [29] and allowed to settle and adhere to the bottom for 30 min at room temperature. The pH was lowered (or not) for 10 min at room temperature to the indicated value (pH 5.7 unless stated otherwise, using an exchange solution consisting of 100 mM NaCl, 1.5 mM KCl, 2.5 mM MgCl_2_, 2.5 mM CaCl_2_, 20 mM MES), the solution was then reneutralized to pH 7.2 by an exchange of solutions, and the temperature raised to 37°C. After this reneutralization for the indicated time, generally 2 h, fusion was scored as a function of time by the transfer of calcein into target cells, as described [64]. For fusion experiments utilizing IVA HA as control, the expressed HA in effector cells were was cleaved into HA1-HA2 subunits with trypsin, as previously described [28].

### Lipid dye mixing

To label effector cells, ~ 2x10^6^ cells/ml were incubated with 10 μM DiO for 30 min at 37°C. The day before an experiment, target cells were split and plated on glass cover slips placed in culture dishes so as to allow convenient transfer. These target cells were labeled by 100 μM DiI for 30 min at 37°C. Labeled effector cells were thermolysin treated (200 μg/ml) and added above labeled target cells. Binding was allowed to occur for 40 min at room temperature before washing out unbound effector cells. The solutions bathing the cover slips (one cover slip per culture dish) was acidified to the indicated pH for 10 min at room temperature, and the culture dish placed in a 37°C incubator for 2 h. Cells were detached from cover slips by adding 10 μg/ml trypsin to a divalent-free solution containing 0.5 mM EDTA for 10 min at room temperature, followed by vigorous, repeated pipetting to dissociate bound (i.e., neither hemifused or fused) cells. Colocalization of the two lipid dyes was monitored by flow cytometry (Guava Easy Cite, Guava Technology, Millipore), using two channels emission, one for each dye (515 nm for DiO and 560 nm for DiI; both excited by a 488 nm laser). The same protocols were followed for mock-transfected COS7 cells; this data was subtracted from data obtain for COS7 cells expressing EBOV GP to obtain percentages of fused cells.

### Production of sNPC1 and measurements of NPC1 expression on the cell surface

The domain C of NPC1 was cloned into a pGEX-4T1 vector that had a GST tag on the N-terminus (GE Healthcare Life Sciences, Pittsburgh, PA). The expression of fusion protein was induced in *E*. *Coli*. by IPTG (0.5 mM) and purified by glutathione sepharose 4B (GE Healthcare Life Sciences). Protein was quantified by a Bradford assay and used for cell-cell fusion and for measurements of cleaved GP. The expression of NPC1 on 293T cell surfaces was determined by using anti-NPC1 (against N-terminus 34–174 aa; LifeSpan BioSciences).

*Determination of EBOV GP cleavage*. HEK293T cells were transfected with EBOV GP or GP^furin^ in the presence or absence of a plasmid that encodes furin (kind gift of Paul Bates, University of Pennsylvania). Transfected cells were detached by PBS containing 5 mM EDTA. One portion (1 million cells) was used to measure the cleaved GP by incubating cells with 2 μg sNPC1 for 2 h on ice, followed by adding a mouse monoclonal anti-GST antibody; the fluorescence signal was quantified by adding a FITC-conjugated secondary anti-mouse antibody using flow cytometry. Another portion of the transfected cells (also 1 million) was used to measure the total GP expression on the cell surface, using an anti-FLAG antibody. The total GP, as determined by mean fluorescence intensity (MFI), was used to determine the percentage of GP on the plasma membrane that was cleaved: the amount of cleaved normalized by total GP. Cleavage of GP^furin^ in cell lysates was determined by Western blotting using anti-FLAG or an anti-GP1 antibody.

### Viral infection

Production of MLV retroviral pseudotypes bearing EBOV GP and viral infection were as described previously [66]. Briefly, 293 GP/LAPSN packaging cells stably expressing MLV Gag-Pol and alkaline phosphatase (AP) were transfected with plasmids encoding the EBOV GP or mutants, and the viruses produced were used to infect HTX cells (a subclone of HT10180). Viral infectivity was determined by counting AP^+^ foci 72 h after infection.

### Proteolysis, altering intracellular trafficking, and inhibition of protein synthesis

We proteolytically determined the stages of fusion at which EBOV GP was proteinase K-sensitive by treating cells with 0.2 mg/ml proteinase K for 20 min, and maintaining or removing it as indicated, at various points in our protocol (S1 Fig). We cleaved EBOV GP with thermolysin and used our standard protocol (a 10-min pH 5.7 pulse), incubated the cells for 2 h at pH 7.2, and then measured fusion. All experiments were performed in parallel on the same days; as controls, proteinase K was not employed and extents of fusion were measured. Brefeldin A (50 μM) was used to inhibit anterograde protein trafficking as described in the experiments of S4 Fig.

### Kinetics of fusion and pore enlargement determined by dye spread

We determined the latency between lowering pH and fusion by using cells mixed within a tube, placed over poly-lysine coated cover slips within a culture dish maintained at 10°C and allowed to settle for 30 min. The pH was lowered to the indicated value for 10 min at room temperature through an exchange of solutions. The cover slips were then transferred into dishes at 37°C, neutral pH, for indicated times. The time of transfer is defined as time = 0. For thermolysin-treated cells, the effector cells were treated prior to binding to target cells. The extents of fusion were quantified at varied times by spread of calcein.

We used the rate of accumulation of calcein into the target cell and its depletion in effector cells to access the size of the fusion pore as a function of time [62]. The fluorescence of both effector and target cells was proportional to calcein concentration, as verified by the procedure detailed in [62].

### Neutralizing intracellular compartments

NH_4_Cl (10 mM unless otherwise noted) was added to the bathing solution and maintained throughout the course of an experiment, including during any low pH pulses, to obtain neutralization. The same procedure was used with chloroquine (100 μM unless stated otherwise) or BafA1 (25 or 100 nM) to cause endosome neutralization. The ionic and buffering contents of the NH_4_Cl-containing low pH solutions were pre-adjusted to the required pH so as to maintain osmotic strength at 290 mOsM during low pH pulses.

### Immunostaining

To monitor EBOV GP expression and its recovery after proteinase K treatment, cells were treated with 0.2 mg/ml of the protease for 20 min at room temperature, and the staining protocol now described was used immediately or after cells were maintained for 3 h in DMEM at 37°C, as indicated. EBOV GP-expressing COS7 cells were transferred to 15 ml tubes and incubated for 1 h at room temperature with a primary antibody (human anti-EBOV GP-KZ52 stock at 1.3 mg/ml, IBT Bioservices, Gaithersburg, MD) that was diluted 1:200 in PBS^++^ that was supplemented with 10% fetal bovine serum. After three washes with the 10% FBS-PBS^++^ solutions, a secondary FITC-conjugated goat anti-human antibody (Fisher Scientific) was added at a final concentration of 100 μg/ml and maintained for 45 min at room temperature in the dark. After cells were washed twice, they were added to 8-well slides that had been treated with poly-L-lysine (M.W. 70,000–150,000, Sigma Aldrich, St. Louis, MO). The cells were then fixed for 20 min at room temperature with 2% paraformaldehyde, and washed twice with DMEM.

### Patch clamp

Pore size over time was also quantified by using patch clamp time-resolved admittance measurements [64], often referred to as capacitance measurements. Fusion was promoted by a 10-min pH 5.7 pulse at room temperature. Because the fusion pore took considerable time to form, and a seal between the patch pipette and cell could only be maintained once solutions and temperature were established, temperature was raised to 37°C for ~15 min before attempting electrical measurements. (This procedure reduced the time between establishing the seal and fusion pore formation). The cover slip was then placed in a temperature-controlled chamber on a microscope stage, and the seal established. The external solution consisted of 135 mM *N*-methyl-glucamine aspartate-5 mM MgCl_2_-2 mM HEPES (pH 7.2); the solution within the patch pipette was 135 mM cesium glutamate-5 mM MgCl_2_-5 mM BAPTA [1,2-bis(*o*-aminophenoxy)ethane-*N*,*N*,*N*′,*N*′-tetraacetate]-10 mM HEPES (pH 7.2). To electrically characterize fusion pores created by IAV HA, ASLV Env, and HIV Env, we used cell lines that stably express the fusion protein and target cells that stably express the cognate receptor: HAb2 cells that express IAV HA as effectors and 293T cells as targets [64]; NIH 3T3 EnvA cells that express ASLV Env and 293T TVA cells as targets [64,67]; and TF228 cells that express HIV Env and Hela T4 cells as targets [29].

### Statistics

Pair-wise Student t-tests were used to compare the outcome of a manipulation on fusion as compared to the control. In figures, unless otherwise indicated, a single asterisk (*) denotes p < 0.05, two asterisks (**) denotes p < 0.01, and three asterisks (***) denotes p < 0.001.

## Supporting Information

S1 Text Supporting information. Consequences of membrane trafficking of EBOV GP on cell-cell fusion.
(DOCX)

S1 Fig Dose-response curves for inhibition of EBOV GP-mediated fusion by 3.47 (A) and KZ52 (B), plotted semi-logarithmically. (TIF)

S2 Fig Proteinase K treatment demonstrates that protein synthesis and trafficking of EBOV GP to the cell surface contributes to the extents of fusion. **(A)** The periods in which proteinase K (PK, 200 μg/ml) was present are marked in the schematic protocol, and numbers correspond to bar numbers below. Regardless of whether PK was present prior (bar 2) or subsequent (bar 3) to treating effector cells with thermolysin, the presence of PK virtually eliminated fusion. Similarly, adding and then maintaining PK to effector cells as they were bound with target cells led to greatly reduced fusion (bar 4). But adding PK immediately after the low pH pulse (bar 5) hardly affected fusion. **(B)** EBOV GP-mediated fusion recovered over time after proteinase K treatment: Left-hand bars of each pair denote that a pH pulse was not applied; a pH 5.7 pulse was applied for the right hand bars. Adding proteinase K and washing out immediately prior to thermolysin treatment virtually abolished fusion (first set of two bars). Allowing 2 hr between proteinase K removal and thermolysin treatment restored most of the fusion (second set of bars). Waiting 3 h completely restored fusion (third set of bars).
(TIF)

S3 Fig Immunostaining of cells demonstrates recovery of cell surface EBOV GP after proteinase K treatment. Left hand panels of each pair show confocal images of FITC fluorescence alone; right hand panels show fluorescence and cells in differential interference contrast. An anti-EBOV GP antibody (KZ52) was used for staining EBOV GP. A secondary FITC-labeled antibody was used to immunostain. **(A)** Immunostaining showed that mock-transfected cells did not react with the antibody. **(B)** Cells transfected with EBOV GP did show significant staining (upper right images). **(C)** The staining protocol was used without delay after treating cells with PK. **(D)** Maintaining the cells for 3 h in DMEM at 37°C before immunostaining. **(E)** The effect of PK treatment on EBOV GP expression was assessed using Volocity imaging software (Perkin Elmer). Integral fluorescence per field (3 image fields per datum point) was calculated after subtracting the fluorescence background determined from the mock-transfected images. This quantification shows that expression of EBOV GP was greatly reduced by the proteinase K treatment and significantly recovered after the protease was absent for 3 h. This demonstrates that the EBOV GP expression levels were steady over time.
(TIF)

S4 Fig Inhibitors of trafficking show that EBOV GP is dynamically exchanged between plasma and intracellular membranes. The presence of Brefeldin A (BFA, 50 μM) at all points of the fusion protocol that utilizes thermolysin-treated effector cells and a pH 5.7 pulse reduced fusion greatly (bar 2) compared to the control (bar 1, BFA was not included). Washing out BFA and immediately treating effector cells with thermolysin led to greater fusion (bar 3). Waiting 30 min after the washout before thermolysin treatment led to fusion (bar 4) comparable to control. Adding and maintaining BFA after binding effector and target cells, but before applying a low pH pulse led to substantially reduced fusion (bar 5). Applying BFA after the low pH pulse led to less fusion than the control (bar 1), but to greater fusion than when the drug was added prior to the low pH pulse (bar 6, extent of fusion higher than for bar 5). Thermolysin was used to cleave EBOV GP just prior to measuring fusion for all conditions of, allowing meaningful comparisons.
(TIF)
