# Supplementary material for: Induction of Cell-Cell Fusion by Ebola Virus Glycoprotein: Low pH Is Not a Trigger
Source: PLoS Pathog. 2016 Jan 5;12(1):e1005373. doi: 10.1371/journal.ppat.1005373 (PMC4711667; doi:10.1371/journal.ppat.1005373)
Supplement: S1 Text — Consequences of membrane trafficking of EBOV GP on cell-cell fusion. (DOCX) [file ppat.1005373.s001.docx]

**Supporting Information**

*Consequences of membrane trafficking of EBOV GP on cell-cell fusion*.

Proteinase K (PK) treatment. We used digestion of cell surface EBOV GP by PK to show that cell-cell fusion was strongly dependent on membrane trafficking of GP. Incubating effector cells with PK either before (Fig. S2A, bar 2) or after (bar 3) thermolysin treatment each led to minimal extents of fusion, showing that cleaved and uncleaved GP is effectively degraded by PK. Adding (and then maintaining) PK prior to the low pH pulse, but after binding effector and target cells, led to significantly less fusion after reneutralization (bar 4) than did the control (bar 1). The addition of PK immediately after the low pH pulse led to the same extent of fusion (bar 5) as did the control (bar 1), strongly indicating that low pH induced conformational changes in GP (through cleavage by cathepsins). Insensitivity of fusion to PK after, but not before, an acidic pulse has previously been found for IAV HA-induced fusion [30].

We found that the elimination of fusion by PK was reversed over time (Suppl. Fig. 2B). Effector cells were incubated with PK, washed, and then treated with thermolysin. Immediately carrying out our fusion protocol led to negligible extents of fusion, independent of whether a low pH pulse was employed (first set of two columns; bar 1 in absence of pulse, bar 2 with low pH pulse). If effector cells were allowed to recover for 2 h at 37°C before thermolysin treatment and fusion then initiated, extents of fusion were comparable to those recorded when PK had not been used. This was the case in the absence of a low-pH pulse (bar 2 of second set of columns) or with a pH-5.7 pulse (bar 2 of second set of columns). Increasing the time of recovery to 3 h after removing proteinase K led to extents of fusion comparable to those in which PK was never added (compare bar for low pH pulse after a two or three hour recovery (Suppl. Fig. 2B) to bar 1 of Suppl. Fig. 2A). The high level of fusion with recovery after protein degradation was likely due to new copies of EBOV GP that reached the cell surface.

We therefore used immunofluorescence to verify that cycling of EBOV GP was the reason fusion was restored over time. The fluorescence of effector cells expressing EBOV GP is shown (Suppl. Fig. 3B). Background fluorescence for mock-transfected cells was negligible (Suppl. Fig. 3A). Incubating cells with PK under the same conditions as used for fusion experiments and immediately staining led to a markedly reduced fluorescence (Suppl. Fig. 3C and left bar of Suppl. Fig. 3E). This shows that the PK had destroyed the integrity of EBOV GP on the cell surface. The fluorescence levels were comparable to control when waiting 3 h after treating cells with PK (including the washout) before performing immunostaining (Suppl. Fig. 3D and right bar of Suppl. Fig. 3E). This demonstrates that the expression level of intact EBOV GP recover over time after PK treatment, and accounts for the observed time course of cell-cell fusion subsequent to removing PK. In conclusion, cell-cell fusion is dynamic, subject to EBOV GP retrieval from and delivery to the cell surface by normal cellular processes during the course of a fusion experiment.

Brefeldin A (BFA) treatment. Effector cells were incubated with the inhibitor (30 min at room temperature) prior to our standard thermolysin treatment, then bound to target cells, and fusion was induced by pH 5.7. BFA was constantly maintained to inhibit delivery of EBOV GP to the cell surface throughout an experiment (Suppl. Fig. 4). The treatment led to significantly reduced fusion (Suppl. Fig. 4, bar 2) compared to the control (bar 1) in which BFA was not present. This reduced fusion is consistent with removal of thermolysin-treated GP from the cell surface and the blockage of GP (both uncleaved and cathepsin-cleaved) transport from intracellular stores to the plasma membrane during the time course of our experiments: BFA prevented the replenishment of GP to the plasma membrane. In contrast, washing out BFA before thermolysin treatment led to a significantly restored extent of fusion (Suppl. Fig. 4, bottom panel, bar 3). Allowing the effector cells to recover for an additional 30 min after the BFA washout, but before the thermolysin treatment, led to full restoration of fusion (Suppl. Fig. 4, bar 4). After cleaving EBOV GP with thermolysin, adding BFA to inhibit intracellular trafficking, and then binding these effector cells (always maintaining the concentration of BFA) to target cells, low amounts of fusion were observed two hours after a pH 5.7 pulse (Suppl. Fig. 4, bar 5). The reduction in fusion is again consistent with the removal of thermolysin-cleaved GP from the cell surface and the prevention of GP delivery to the surface by BFA. Each process, alone or in combination, would lead to an insufficient density of GP on the cell surface. A greater extent of fusion occurred when the pH 5.7 pulse was applied after adding BFA (bar 6); this is expected because a low-pH pulse induces significant fusion, consistent with activation of cathepsins on the cell surface (see Fig. 2B, thermolysin) before EBOV GP is presumed to be removed from the plasma membranes. Taken together, the results of these experiments clearly indicate that the extents of fusion correlate with the expected amount of cleaved EBOV GP on the cell surface.
