# Supplementary figures and images for: Induction of Cell-Cell Fusion by Ebola Virus Glycoprotein: Low pH Is Not a Trigger
Source: PLoS Pathog. 2016 Jan 5;12(1):e1005373. doi: 10.1371/journal.ppat.1005373 (PMC4711667; doi:10.1371/journal.ppat.1005373)

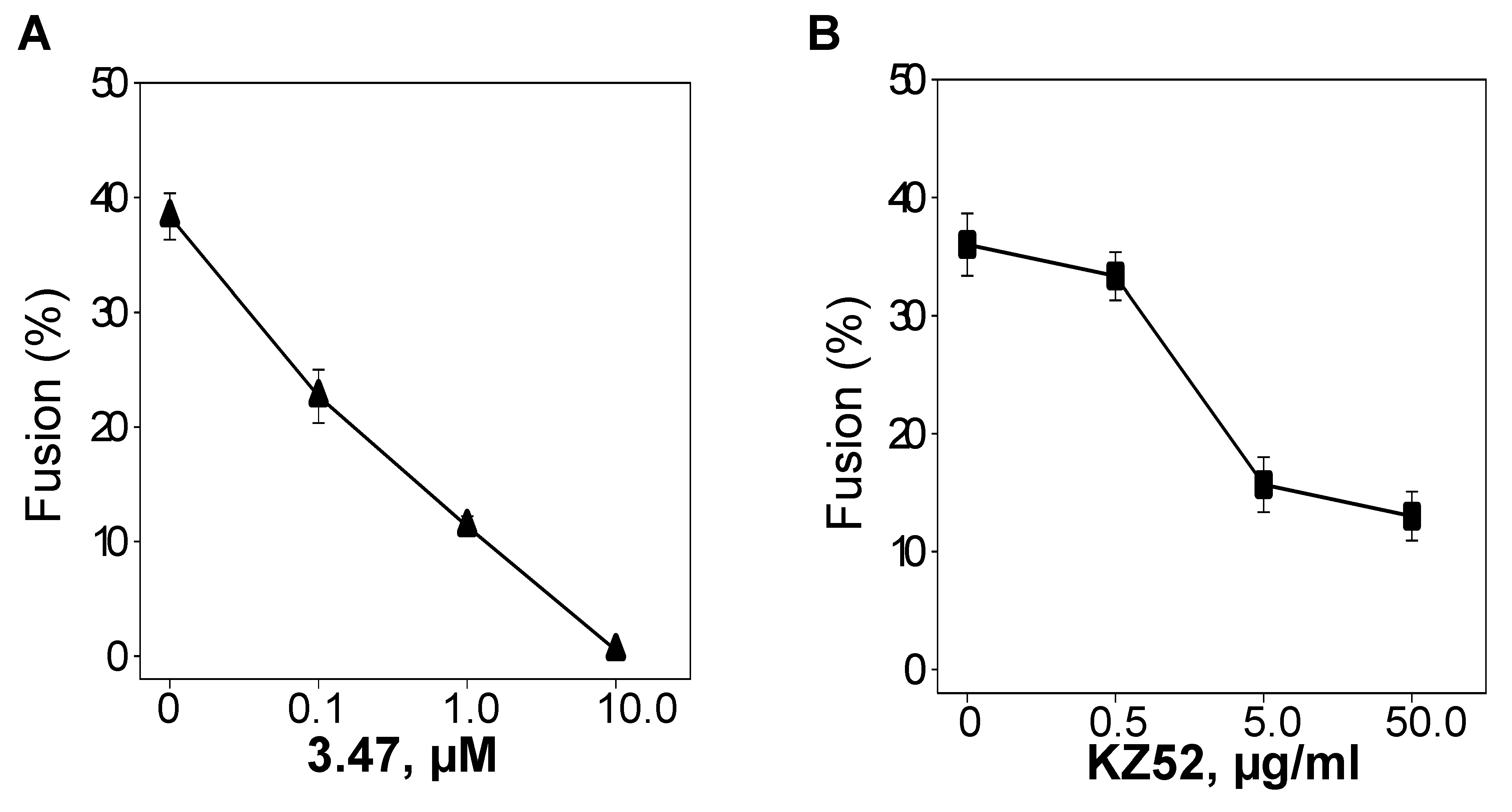

Supplement: S1 Fig — (TIF) [file ppat.1005373.s002.tif]

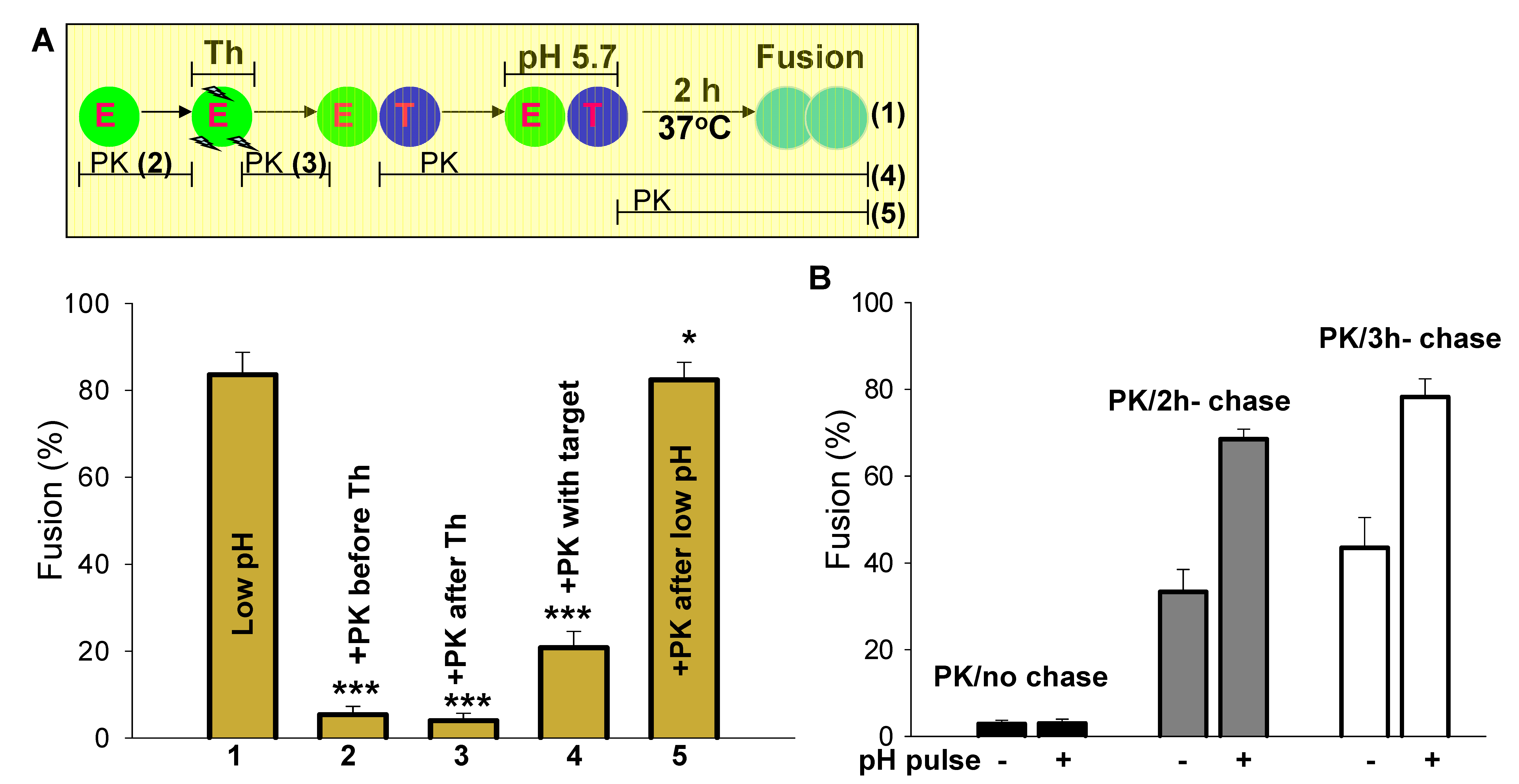

Supplement: S2 Fig — (A) The periods in which proteinase K (PK, 200 μg/ml) was present are marked in the schematic protocol, and numbers correspond to bar numbers below. Regardless of whether PK was present prior (bar 2) or subsequent (bar 3) to treating effector cells with thermolysin, the presence of PK virtually eliminated fusion. Similarly, adding and then maintaining PK to effector cells as they were bound with target cells led to greatly reduced fusion (bar 4). But adding PK immediately after the low pH pulse (bar 5) hardly affected fusion. (B) EBOV GP-mediated fusion recovered over time after proteinase K treatment: Left-hand bars of each pair denote that a pH pulse was not applied; a pH 5.7 pulse was applied for the right hand bars. Adding proteinase K and washing out immediately prior to thermolysin treatment virtually abolished fusion (first set of two bars). Allowing 2 hr between proteinase K removal and thermolysin treatment restored most of the fusion (second set of bars). Waiting 3 h completely restored fusion (third set of bars). (TIF) [file ppat.1005373.s003.tif]

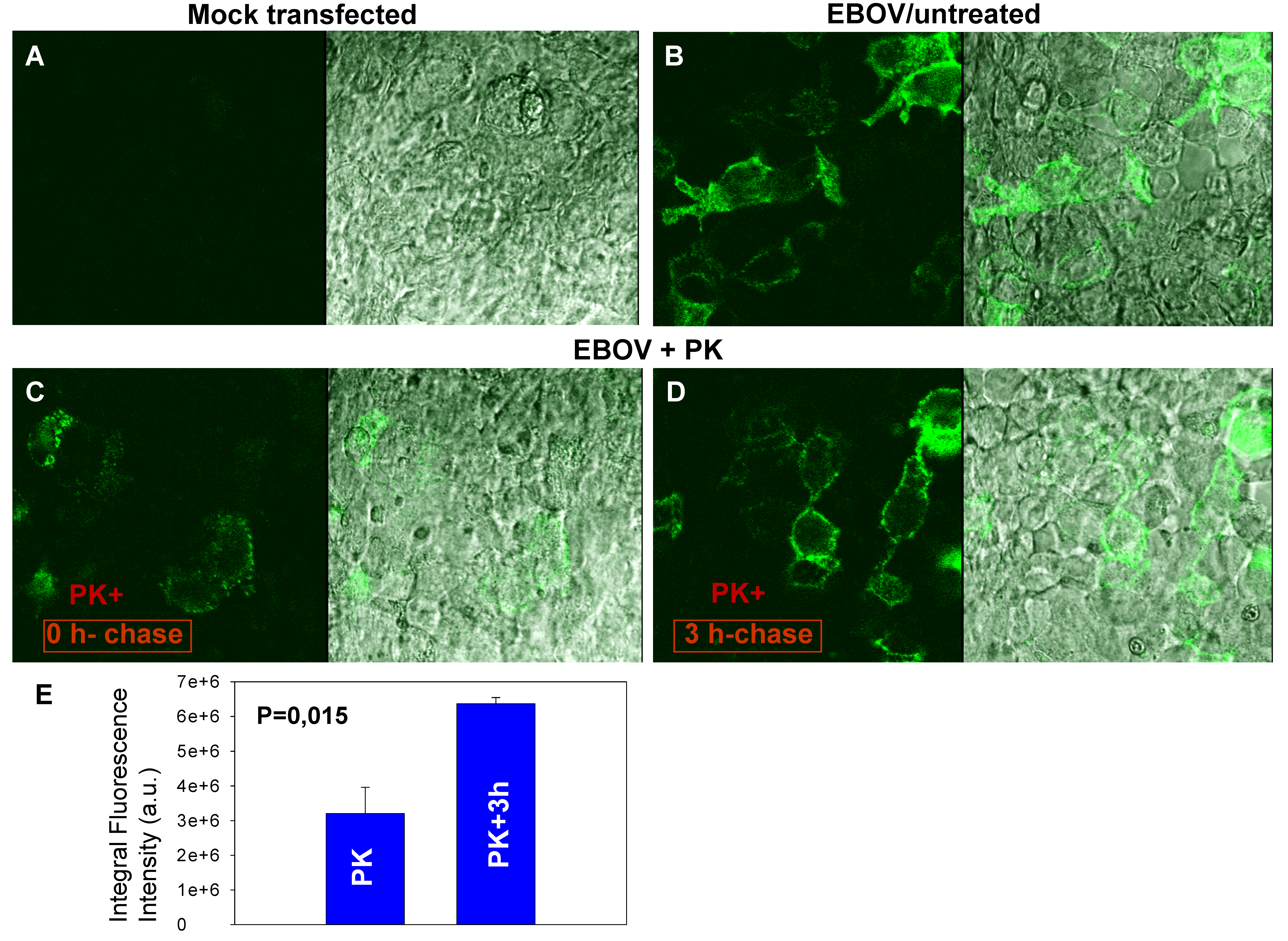

Supplement: S3 Fig — Left hand panels of each pair show confocal images of FITC fluorescence alone; right hand panels show fluorescence and cells in differential interference contrast. An anti-EBOV GP antibody (KZ52) was used for staining EBOV GP. A secondary FITC-labeled antibody was used to immunostain. (A) Immunostaining showed that mock-transfected cells did not react with the antibody. (B) Cells transfected with EBOV GP did show significant staining (upper right images). (C) The staining protocol was used without delay after treating cells with PK. (D) Maintaining the cells for 3 h in DMEM at 37°C before immunostaining. (E) The effect of PK treatment on EBOV GP expression was assessed using Volocity imaging software (Perkin Elmer). Integral fluorescence per field (3 image fields per datum point) was calculated after subtracting the fluorescence background determined from the mock-transfected images. This quantification shows that expression of EBOV GP was greatly reduced by the proteinase K treatment and significantly recovered after the protease was absent for 3 h. This demonstrates that the EBOV GP expression levels were steady over time. (TIF) [file ppat.1005373.s004.tif]

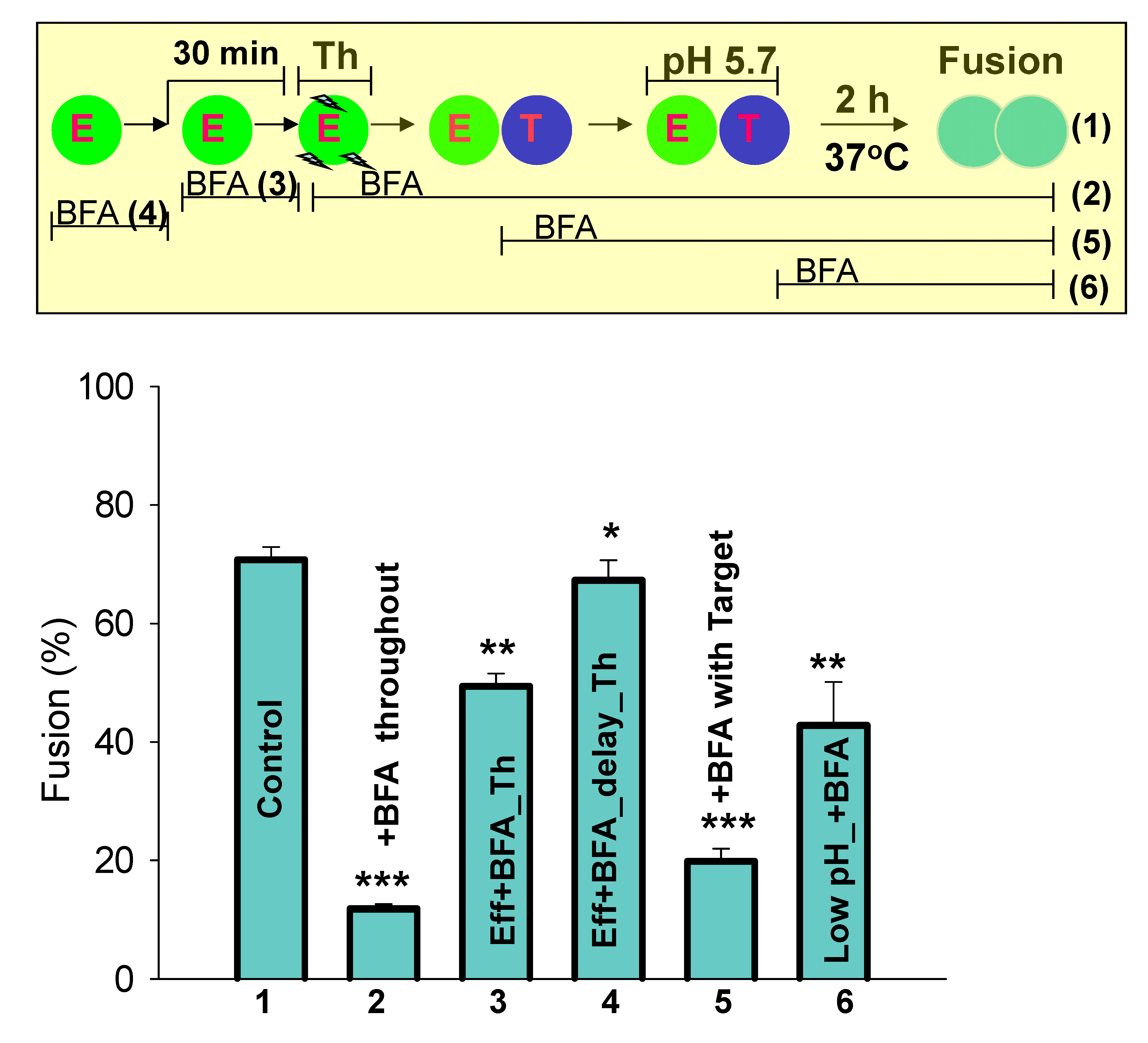

Supplement: S4 Fig — The presence of Brefeldin A (BFA, 50 μM) at all points of the fusion protocol that utilizes thermolysin-treated effector cells and a pH 5.7 pulse reduced fusion greatly (bar 2) compared to the control (bar 1, BFA was not included). Washing out BFA and immediately treating effector cells with thermolysin led to greater fusion (bar 3). Waiting 30 min after the washout before thermolysin treatment led to fusion (bar 4) comparable to control. Adding and maintaining BFA after binding effector and target cells, but before applying a low pH pulse led to substantially reduced fusion (bar 5). Applying BFA after the low pH pulse led to less fusion than the control (bar 1), but to greater fusion than when the drug was added prior to the low pH pulse (bar 6, extent of fusion higher than for bar 5). Thermolysin was used to cleave EBOV GP just prior to measuring fusion for all conditions of, allowing meaningful comparisons. (TIF) [file ppat.1005373.s005.tif]
